# Automated Classification of Humpback Whale Calls Using Deep Learning: A Comparative Study of Neural Architectures and Acoustic Feature Representations

**DOI:** 10.3390/s26020715

**Published:** 2026-01-21

**Authors:** Jack C. Johnson, Yue Rong

**Affiliations:** School of Electrical Engineering, Computing and Mathematical Sciences (EECMS), Curtin University, Kent Street, Bentley, WA 6102, Australia; jackcjohnson2@gmail.com

**Keywords:** marine bioacoustics, humpback whale, convolutional neural network (CNN), vision transformer (ViT), spectrogram, mel spectrogram, MFCC (Mel-Frequency Cepstral Coeffucient), acoustic monitoring, classification

## Abstract

Passive acoustic monitoring (PAM) using hydrophones enables collecting acoustic data to be collected in large and diverse quantities, necessitating the need for a reliable automated classification system. This paper presents a data-processing pipeline and a set of neural networks designed for a humpback-whale-detection system. A collection of audio segments is compiled using publicly available audio repositories and extensively curated via manual methods, undertaking thorough examination, editing and clipping to produce a dataset minimizing bias or categorization errors. An array of standard data-augmentation techniques are applied to the collected audio, diversifying and expanding the original dataset. Multiple neural networks are designed and trained using TensorFlow 2.20.0 and Keras 3.13.1 frameworks, resulting in a custom curated architecture layout based on research and iterative improvements. The pre-trained model MobileNetV2 is also included for further analysis. Model performance demonstrates a strong dependence on both feature representation and network architecture. Mel spectrogram inputs consistently outperformed MFCC (Mel-Frequency Cepstral Coefficients) features across all model types. The highest performance was achieved by the pretrained MobileNetV2 using mel spectrograms without augmentation, reaching a test accuracy of 99.01% with balanced precision and recall of 99% and a Matthews correlation coefficient of 0.98. The custom CNN with mel spectrograms also achieved strong performance, with 98.92% accuracy and a false negative rate of only 0.75%. In contrast, models trained with MFCC representations exhibited consistently lower robustness and higher false negative rates. These results highlight the comparative strengths of the evaluated feature representations and network architectures for humpback whale detection.

## 1. Introduction

Passive acoustic monitoring (PAM) using hydrophones is an established approach in studying marine fauna due to its ability to continuously operate across large spatial scales with minimal ecological impact. However, practical deployment of PAM for ecological monitoring remains constrained by several significant challenges. Foremost, ocean soundscapes are highly complex and dynamic environments where biological, anthropogenic and geophysical sources interact to create immensely variable acoustics backgrounds. The acoustic complexity directly hinders the capability of detection and classification of marine mammal vocalizations. Acoustic datasets collected by PAM can cover hundreds to thousands of hours, meaning that manual review is labour-intensive and time-consuming and fundamentally limiting the scalability of the system [1]. These challenges strongly justify the need for automated solutions to operate reliably across diverse conditions.

Recent advancements in machine learning offer promising solutions to automate the process of detecting and classifying marine fauna sounds [2], yet several key challenges remained unresolved.

### 1.1. Key Challenges and Limitations

#### 1.1.1. Underwater Noise Variability

Underwater noise conditions vary drastically across location, time, and recording equipment, introducing significant variability across data. The increase in anthropogenic activities in marine environments, from operations such as shipping and offshore drilling, has drastically propagated the amount of underwater noise pollution. These sounds are significantly detrimental to marine fauna, in particular the species that rely on sound for communication, navigation, and foraging [3]. The original complex nature of these ocean soundscapes is convoluted by the combination of these additional overlapping signals from various sources, hindering the classification and detection of marine species. Current methods are prone to inaccuracies when differentiating between relevant biological sounds and background noise as critical signals are masked with prevalent feedback [4].

#### 1.1.2. Data Variability, Scarcity and Generalization

The reliability of acoustic data collected from various sources can substantially fluctuate in terms of traits and characteristics which complicates machine learning model training [5]. For example, the performance of CNNs in marine mammal vocal classification is affected by the diverse possibilities of audio sources and background noise [6]. Generalization across various environments presents issues among numerous existing models as they are trained on distinct data sets that do not generalize well with different marine environments and species. This outlines the need to develop new models capable of adapting to varying acoustic conditions [6]. The online availability of labelled datasets for this specific task is minimal, No such dataset could not be sourced for testing. This led to the procurement of unlabelled audio to be manually edited and curated into a labeled dataset, introducing a high risk of bias and error.

#### 1.1.3. Integration of Human Expertise

Integrating human expertise is important to ensure the results and their accuracy are validated by professionals. Developing a workflow that combines automated systems with a level of human review is integral for practical marine monitoring applications [7]. A system developed in mind of a beneficial feedback loop where insights gained by human analysis can be used to further improve learning models should be pursued.

This study explores deep machine learning applications for classifying humpback whale calls using an original dataset. The custom, here-developed convolutional neural network (CNN) and vision transformer (ViT) architectures are evaluated alongside the pre-trained MobileNetV2 model. In a controlled environment two feature extraction methods are compared– the mel spectrogram and the Mel frequency Cepstral Coefficient (MFCC) methods–and several data-augmentation techniques are examined, including noise addition, pitch shifting, time shifting, and filtering. These methods are applied with a modular preprocessing and segmentation pipeline for reproducibility and real-time application.

Modern studies have demonstrated the benefits of passive acoustic monitoring systems, especially for species including the humpback whale. Research shown in ref. [8] examines non-song sounds created by migrating humpback whales and their importance in social interactions. These audio sets, which also include examples of breaching and slapping, can effectively be analyzed by machine learning tools to document and enhance our understanding of whale communication and behaviour. Another study, ref. [9] explores humpback whale call classification and introduces the concept of “subunits” which are comparable to phonemes in human speech. These features are well documented to be classifiable by neural networks, indicating that an MFCC approach may improve the accuracy when identifying whale vocalizations and in further improvements of automated classification systems.

The effects of vessel noise on humpback whale communication networks are investigated in ref. [10] which reveals how this intrusive sound interferes with their communication vocalizations by masking the whale’s natural sounds. This raises alarming implications about the detrimental effect on humpbacks which rely on acoustic signals for social interactions, navigating and mating. Studies show that, in environments saturated with vessel noise, humpback whale low frequency call ranges are reduced from 4 km to 2 km by this interference. This reduction in communication area leads to decreased social interaction between whales, with a reported 50% reduction in interactions, having adverse effects on mating and group cohesion. Whales are also recorded to alter their speed and migration routes to avoid vessels, further disrupting their natural behaviours and social dynamics [10]. The cumulative effects of these vessel noises on whales raise severe implications for population dynamics in all marine species that depend on acoustics in their natural behaviours.

The modern developments in acoustic technologies such as hydrophone arrays and recent signal processing techniques, have drastically improved the capabilities of machine learning applications for marine acoustic monitoring. The further development of these models for marine-based applications increases in demand as the volume of acoustic data grows [11]. The ability to process large datasets according to various marine environments would allow researchers to better identify patterns and trends in marine fauna behaviour, a powerful tool for understanding and managing marine ecosystems.

### 1.2. Objective, Motivation, and Contributions

The objective of this study is to evaluate a range of deep learning architectures to identify an effective model for the real-time detection of humpback whale vocalizations from short audio segments. This classification requires that we developed models that are capable of accurate prediction within tightly constrained temporal windows while maintaining robustness to noise, recording condition variability and natural vocalization diversity. The development of these capabilities is integral to improving the autonomy of PAM systems, increasing understanding between the correlation of acoustic bio-signatures and behavioral patterns, enabling efficient macro ecological surveillance and mitigating risks associated with collisions between sea vessels and marine life [4]. The main contributions of this paper are summarized below.

- Created a high-quality, labeled dataset. A large collection of unlabeled audio sourced from reputable online repositories was manually curated, reviewed, and segmented to minimize annotation error and dataset bias. This resulted in a reliable and domain-appropriate labeled dataset for supervised training.
- Design of a complete data-processing pipeline. A modular preprocessing framework was developed incorporating mel spectrogram and MFCC feature extraction, deterministic segmenting strategies, controlled overlap, and a diverse set of audio augmentation techniques aimed to improve model generalization.
- Comparative evaluation of CNN and ViT architectures. Multiple architectures were trained and tested under matched conditions, including models trained with and without augmentation. A comprehensive suite of performance metrics including accuracy, F1 score, receiver operating characteristic (ROC) curves, precision-recall curves, Matthews correlation coefficient (MCC), and error rate. Thorough analysis is presented to enable rigorous comparison and highlight the capacity of generalization to unseen test data.
- Insightful analysis of feature representations and model behaviour. The study examines the comparative effectiveness of mel spectrograms against MFCCs, the influence of augmentation on model stability, and the architectural differences between convolutional and transformer-based approaches. These findings provide a direction for future research into real-time-deployable autonomous PAM systems and emerging technologies that require reliable the detection of distinctive bioacoustic signatures.

## 2. Materials and Methods

### 2.1. Spectrograms and Variants

A spectrogram represents the frequency spectrum of signals over time. It provides an extensive source of information which is beneficial for identifying and classifying sounds [12]. Spectrogram is generally the primary feature representation tool for audio classification and its effectiveness has been well documented. A spectrograms allow us to change audio classification into image classification, a broader and more prominent area of research. The authors of [13] discusses the use of pretrained ImageNet CNN models for classification tasks, providing evidence that the models are capable of effective learning using spectrograms despite the obvious differences between audio and image data. This evidence indicates that transfer learning from image data sets can produce competitive results for audio classifications, further highlighting that similar methodologies can be utilized in marine acoustic monitoring to enhance detection capabilities. An ImageNet model (MobileNetV2) has been applied in testing to demonstrate capability in this specific study and to compare performance with the custom architectures.

The authors of [14] outlines the use of mel spectrograms, which are a modified spectrograms that are adjusted to a logarithmic scale based on human hearing. They are shown to enhance classification performance for audio event detection when dealing with unknown audio devices. As acoustic characteristics substantially vary across the recording devices used in marine environments, this alternate spectrogram is highly applicable to the outlined challenges. The study explores how mel spectrograms better reflect the nuances of sound events, greatly improving the robustness of machine learning models in real-world scenarios [15].

MFCCs, represent the rate of change in cepstral coefficients over time. This temporal information is crucial for detecting the frequency modulations and rhythmic patterns characteristic of whale calls. The visualization process is standardized in terms of dimensions and color mapping, resulting in spectrograms that are both visually uniform and information dense.

A comparative study between a Mel-Frequency Cepstral Coefficient (MFCC)-based approach and a non-negative matrix factorization (NMF)-based approach [16] demonstrates that the latter may be more robust for audio detection under noisy conditions. The study presents an approach using convolutive NMF to extract spectro-temporal features from audio signals. This method provides temporal context alongside spectral information which is claimed to more accurately detect acoustic events within complex ocean environments. It concludes with results outlining how this approach surpasses the accuracy of traditional MFCC systems, especially in noisy conditions. This indicates the importance of integrating temporal dynamics in the feature extraction process [16].

The authors of [17] introduces a competitor to speech-aligned MFCC; it has a differently scaled frequency window for non-speech audio named gammatone cepstral coefficients (GTCC) approach. GTCCs are biologically inspired features for non-speech audio classifications whereas MFCC is designed with speech recognition in mind. The authors of [17] provides evidence that GTCCs are superior representations of spectral characteristics within non-speech audio signals as they can capture critical details in low frequency ranges.

### 2.2. Data Collection and Preprocessing

To effectively train models, it is critical to conduct competent data collection to ensure the credibility of accuracy. To reproduce any meaningful results and reliable material, the quality of testing and training data is paramount to produce reliable material.

**Figure 1:**
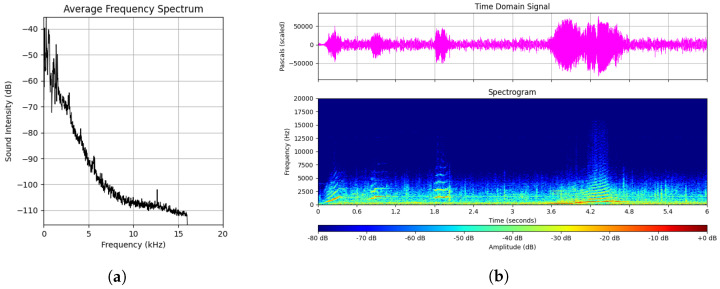
(**a**) A typical sound spectra of a humpback call taken from audio sources, (**b**) six-second pressure (**upper-right**) and its corresponding spectrogram (**lower-right**).

Acoustic monitoring is a method of continuous data collection that can be used on marine fauna, especially in extreme environments where visual observations are difficult. The deployment of passive acoustic listeners (PALs) has been regarded as a proficient tool to gather ambient audio data in the ocean. They are capable of capturing a wide range of frequencies (0–50 kHz) and detect biological sounds by marine animals as well as anthropogenic sources such as ships and sonars [18]. These devices operate without interference to the marine environment, forming an unobtrusive and reliable system for monitoring marine life over extended time periods. Figure 1 shows the typical sound spectra, sound pressure, and spectrogram of a whale call.

After audio data are collected, they must be labeled meticulously to ensure the accuracy and reliability of machine learning applications. As established in ref. [19], data labeling can cause bottlenecks in machine learning workflows, especially in novel implementations with limited training data. Techniques to label data include crowdsourcing and active learning. Crowdsourcing provides a collection of labels–from a diverse group of participants–which is well suited for large-scale monitoring applications [19]. Active learning methods assist in the prioritization of data points to be labeled based on their potential performance impacts on a model.

Implementing real-time data reporting systems, such as the Greece-based Poseidon system, demonstrates the capability of immediate analysis and responsive to acoustic events [18]. The PAL system transmits data in three-hour intervals, which provides time-sensitive information on marine mammal activity and current environmental conditions. The systems provide information for adaptive management strategies in marine conservation by supplying recent data for quick adjustments [18].

Sourcing data from preexisting audio repositories to produce custom compositions will be essential for this research scope. Various open-source libraries exist online where samples can be collected, including organizations such as the AODN (Australian Ocean Data Network) and internet repositories hosted by BBC, Watkins and Ocean Alliance.

#### Implementation

Audio files are collected from an array of online sources including BBC, Watkins, Ocean Alliance, and the Australian Ocean Data Network [20,21,22,23]. The total size of the original audio utilized is 8 GB. To ensure consistency, all tracks are standardized using the Audacity software version 3.7.7 to mono channel, 44.1 kHz sample rate, 16-bit depth precision and lossless compression (bit rate approximately 700 kbps). This preprocessing step reduces variation due to different recording hardware, mitigates channel imbalance and filters artifacts such as out-of-band noise and compression noise.

To develop an algorithm that can detect marine life presence, the temporal analysis window must be sufficiently small to allow rapid prediction updates while capturing the full structure of humpback whale vocalizations. A time window of six seconds is selected, with three seconds of overlapping between consecutive audio segments. This method provides several benefits: (1) the larger window allows the algorithm to preserve call structure for accurate prediction; (2) acoustic predictions are refreshed every three seconds, supporting real time deployment; (3) it allows multiple opportunities to detect the same event, reducing missed call probability.

The time window introduces an issue with collected audio. When a window of 6 s or greater contains no whale call, the spectrogram produced would require classification as ‘no call’. To prevent incorrect labeling, the audio sources acceptable to pass through the pipeline have had considerable time taken to be manually assessed, curated and clipped. The clipped audio is repurposed as ‘no call’ samples and re-categorized in the dataset as such. All spectrograms were generated from audio resampled to 22,050 Hz, with each 6-second segment containing

6×22,050=132,300samples.

A hop length of 2646 from the following equation was used, producing exactly 50 time frames per spectrogram

$$\mathrm{hop\_length}=\frac{132,300}{50}=2646.$$

Spectrograms were computed using the following parameters:

- **Sampling rate:** 22,050 Hz.
- **FFT size:** 2048.
- **Hop length:** 2646.
- **Window function:** Hann window.
- **Number of mel bins:** 125 (image height).
- **Maximum frequency:** 10 kHz.
- **Amplitude scaling:** log-power spectrogram.
- **Pre-emphasis:** none applied.
- **Normalization:** image standardization applied during model preprocessing.

**Figure 2:**
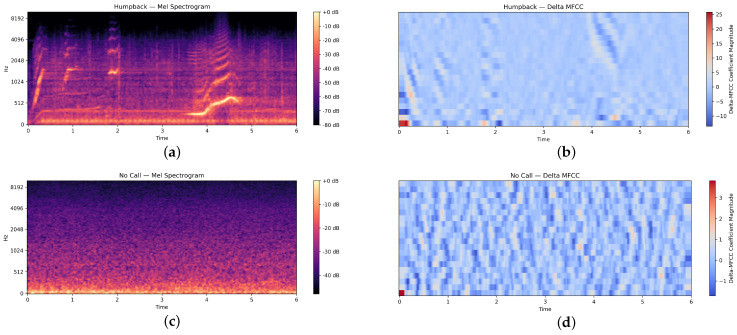
Comparison of humpback call and no-call examples using two feature representations. (**a**) humpback call–mel spectrogram, (**b**) humpback call–Delta-MFCC, (**c**) no-call–Mel spectrogram, (**d**) no-call–Delta-MFCC.

These parameters ensured consistent time–frequency resolution across the entire dataset. MFCC representations were computed using the same sampling rate, hop length, and windowing configuration to maintain comparability across feature domains. A visual example of the feature representations are provided in Figure 2 below, these are scaled up for visibility.

Construction of the training, validation, and test datasets were heavily influenced by the characteristics of the raw audio sources. Due to the origination from different audio repositories, files vary significantly in duration leading to uneven quantities of 6 s segments across classes. Segmenting all files before performing the random split introduces severe data leakage, since temporally adjacent windows from the same recording are distributed across multiple split categories.

This issue was addressed by splitting at the source, allocating complete data files to training, validation, and testing before segmentation. This prevents the temporal leakage and artificial inflation of performance metrics; however, it introduces variability in the resulting dataset sizes. Consequently, the relative ratio of “hump” and “no call” samples varies across the the subsets.

To prevent dataset leakage–where overlapping segments or temporally adjacent segments from the same audio file are organized across different splits–the dataset is structured based on source files. Four datasets were produced with a training set of 70% of sources, a validation set of 15% of sources and a test set of 15% of sources. This method is applied to mel spectrogram and MFCC datasets with and without augmentation.

### 2.3. Data Augmentation

The cogency of models can be hindered by the limited availability of labelled training data, a crucial resource for training reliable classifiers. This issue can be addressed by using prudent data augmentation strategies that enhance the size and diversity of training datasets.

Data augmentation creates variations in the existing data without modifying them beyond their label’s definition. The nature of this change is of paramount importance for audio classification tasks where the high variability of sound recordings can lead to overfitting if the model is trained with a limited dataset. As discussed in ref. [24], data augmentation does not simply increase the amount of training data, but generates a healthy level of variability to help models better generalize with unseen data.

The methods commonly used to augment image data cannot be applied directly to a spectrogram to produce accurate predictions as the image alteration is too detrimental to the nature of the sound represented by spectrogram. Rotating, flipping and random distortion/stretching do not linearly translate to the way the sound representation in the spectrogram is being augmented. This leads to technique development to pursue methods that directly augmented the sound rather than the spectrogram. Studies such as ref. [25] advocate for these methods in which variations are applied to the audio clips before the spectrogram image conversion. The techniques suggested by ref. [25] ensure that the physical meaning of the audio signals is retained which allows the model to learn the relevant features from training data. The results using these methods show exemplary performance in environmental sound classification tests, where high accuracy results are achieved using the datasets ESC-10 and Us8k.

**Figure 3:**
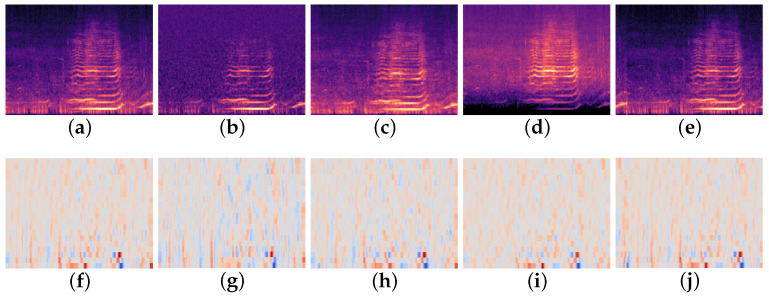
Visual comparison of original and augmented audio samples using mel spectrograms (Mel) and delta MFCC representations. (**a**) original–mel; (**b**) noise–mel; (**c**) pitch–mel; (**d**) highpass–mel; (**e**) shift–mel; (**f**) original–Delta-MFCC; (**g**) noise–Delta-MFCC; (**h**) pitch–Delta-MFCC; (**i**) highpass–Delta-MFCC; (**j**) shift–Delta-MFCC.

Various augmentation techniques are explored, each with strengths and weaknesses. In [26], the traditional methods of adding/removing noise, pitch shifting and time stretching are discussed and shown to increase the robustness of audio classification models [27]. Additionally, methods such as SpecAugment, a technique of masking time and frequency on spectrograms, show beneficial results for improving classification accuracy. This method specifically utilizes the two dimensional nature of a spectrogram to produce informative training samples. Figure 3 demonstrates these effects of data augmentation on the mel spectrogram and MFCC feature representations.

Ensemble approaches that use a combination of multiple classifiers trained on augmented data demonstrate improved performances over individual models [28]. It is discussed in ref. [28] how various CNNs are trained on original and augmented datasets, which have improved recognition capabilities for animal audio classifications. Additionally, the strategy of class conditional data augmentation, in which specific augmentation is tailored for individual classes can further improve model accuracy by considering unique characteristics present in different sound categories [29]. It is demonstrated that data augmentation is a valuable strategy for training models to detect and classify data collected by marine-fauna-monitoring systems. By exploring a variety of augmentation approaches, research can be conducted to improve the integrity and accuracy of future models, allowing improvements in classifying marine audio.

#### Implementation

To increase the robustness of the model and reduce overfitting, several audio augmentation techniques are utilized [28]. Each six-second audio clip undergoes one or a random combination of the augmentations based on a series of probabilities for each augmentation to occur, which helps to simulate real world examples of variability. The techniques that are used include: noise addition, which introduces background noise. Pitch shifting, which alters the frequency of calls slightly to mimic probable whale call variations. Time shifting that adjusts timing of whale calls to produce different time frame possibilities. Then filtering, which is the application of high-pass and low-pass filters.

To improve data diversity and increase generalization capability under realistic marine conditions, a stochastic audio augmentation pipeline is applied to each 6 s sample (training set only). Each augmentation operator is assigned an independent probability of 50% to be applied to an audio sample. To ensure each augmented instance receives some form of variation the process repeats until at least one augment is applied. This preserves randomness without allowing unaltered audio to reach the augmented dataset. The augmentation process is implemented using the python package Audiomentations and consists of the following operators.

**Gaussian Noise:** Gaussian noise was added with a standard deviation between 1 and 1.5% of the signal’s full amplitude. This simulates low level ambient disturbance such as ship traffic, hydrodynamic motion and environmental background noise.

**Pitch Shift:** The pitch of the signal was randomly shifted between −12 and +12 semitones. This replicates natural variability in humpback whale vocalization frequency and encourages the model to learn structural rather than absolute frequency features.

**High Pass Filtering:** A high pass filter with a cutoff frequency randomly selected between 1 and 2 kHz was applied. This mimics filtering artifacts inherent to hydrophone instrumentation and suppresses low frequency interference while preserving harmonic components.

**Time Shift (Roll-over Enabled):** Signals were shifted forwards or backwards by up to ±3 s with rollover enabled. This imitates timestamp misalignment, inconsistent call onset timing, and the natural temporal variability in marine mammal vocal behaviour.

To improve processing times to compensate for computational feasibility on CPU hardware, augmentation is performed once beforehand to produce a dataset as opposed to being reapplied for each training process. Each original audio sample produces five independently augmented variants, drastically diversifying dataset variability and enabling more robust model training.

### 2.4. Final Curated Datasets

The number of 6 s recordings used in the datasets is shown in Table 1 and Table 2 below.

Due to the method of audio collection, the length of audio tracks is not balanced across categories and cannot be separated after being segmented on account of data leakage across the dataset split. To prevent the categories from becoming imbalanced, excessively long files are cut off to reduce bias. The method of source splitting has resulted in the ratio variations across datasets.

### 2.5. Computational Setup

Model training and evaluation were undertaken on a CPU workstation, while GPU acceleration is typically recommended for deep learning tasks, the model configurations, dataset size and computational requirements for this study were manageable on a high performance desktop CPU. Certain drawbacks were observed, notably in ViT training due to computational complexity necessary to complete the training tasks. The specifications are as follows:

- **CPU:** AMD Ryzen 7 5600X3D
  – 8 cores / 16 threads.
  – 3.4 GHz base clock.
  – 4.5 GHz boost clock.
  – Large L3 cache (3D V-Cache).
- **Memory:** 32 GB DDR4 RAM - 3200 MHz.
- **Storage:** Samsung 970 EVO Plus 1 TB NVMe M.2 SSD.

Although CPU performance was adequate for the tasks completed in this study, future work would benefit from dedicated GPU performance. A machine-learning-focused workstation would enable greater complexity hyperparameter use and deeper transformer variants.

### 2.6. Neural Network Architecture (CNN)

**Figure 4:**
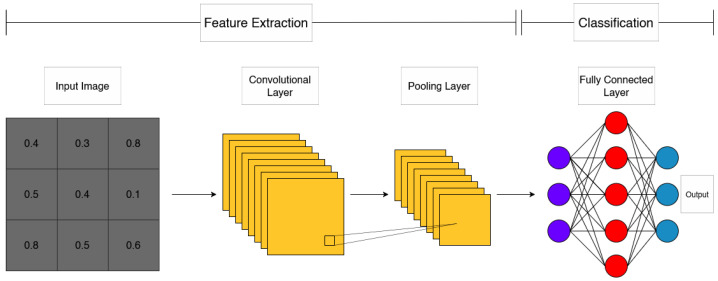
Architecture of a CNN.

CNNs are a class of deep learning models that are highly effective at processing data which consist of grid-like topology such as images. CNN architectures are composed of an intricate mesh of layers that apply convolutional operations to the input data. Each layer applies a set of filters that slide over input data, which creates feature maps. These filters are developed to detect notable features including edges, textures, and patterns [30]. Following convolution operations, an activation function is used to introduce non-linearity. A common function to apply is the rectified linear unit (ReLU), which either outputs a direct input when positive or a zero when negative. Pooling layers are used to reduce the spatial dimensions of feature maps, this greatly reduces computational load and minimizes overfitting. A common technique used is max pooling, which only utilizes the maximum value from a set of values in a select feature map. After multiple convolutional and pooling layers, the final procedure occurs through fully connected layers. In these layers, every neuron in one layer is connected to every neuron in the next layer. This connection map allows the network to make the final prediction based on features extracted in the previous layers [31]. Figure 4 shows the architecture of a CNN.

The design of a CNN automatically learns to extract features from input data in the training process. Earlier layers generally learn the lower level features, while deeper layers learn complicated features such as shapes or specific objects [7]. This hierachical structure is beneficial for image classification tasks. Training a model with a CNN occurs by propagating data forwards and backwards throughout the network. The first forward propagation through the network produces an output, which is compared to defined labels using a loss function. Following this is the backpropagation, where the loss passes back to update the weights of filters and neurons with an optimization algorithm [3]. A common algorithm used for this process is stochastic gradient descent (SGD), which optimizes the weights to minimize the loss function. As the overall process contains an immense amount of decisions, it is a common occurrence for models to ‘overlearn’ the data set they are training on. This is known as overfitting, and various steps can be taken at all stages to reduce the issue. Mitigation techniques lie in regularization which involve batch normalization, dropout and data augmentation [30].

Recent developments in deep learning have substantially advanced the capabilities of acoustic monitoring. CNNs in particular are a powerful tool capable of detecting and classifying in acoustic environments. Many applications and studies have been conducted outlining their effectiveness; for example, in ref. [7] where a CNN is shown to successfully predict the vocalizations of fourteen forest birds and mammals with precision exceeding 90%. In the marine related study [3], a CNN approach for classifying diverse ocean noises achieved an overall accuracy of 96.1%.

Comparative studies have highlighted advantages of deep learning over traditional methods in environmental sound prediction [26]. Deep learning consistently outperforms conventional classifiers including Gaussian mixture models and support vector machines, most notably with large feature sets. In a thorough study on the IEEE DCASE challenge dataset, exemplary classification accuracy was shown using CNNs, with the models implementing previously discussed MFCCs and spectrograms achieving the best results [32].

#### 2.6.1. Application

The model has been iteratively improved using hyperparameter adjustments and inclusion of additional features to curate a custom convolutional neural network that achieves promising results. Both models use 10 epochs, a batch size of 128 and a training/testing split of 70/30. The model begins with an input layer accepting images of shape 125 × 50 × 3. This corresponds to 125 mel bins (height), 50 time frames (width), and 3 colour channels (RGB).

#### 2.6.2. CNN Architecture

Mel spectrogram and MFCC are tested on the same architecture composed of four convolutional layers followed by a fully connected classifier. This provides a controlled testing environment focusing on the capability of each feature representation without architecture bias.

##### Convolutional Block 1

- **Conv2D:** 16 filters, 6×6 kernel, tanh activation.
- **MaxPooling2D**.
- **Batch Normalisation**.
- **Dropout:** 0.5.

##### Convolutional Block 2

- **Conv2D:** 32 filters, 4×4 kernel, ReLU activation.
- **MaxPooling2D**.
- **Batch Normalisation**.
- **Dropout:** 0.4.

##### Convolutional Block 3

- **Conv2D:** 64 filters, 3×3 kernel, ReLU activation.
- **MaxPooling2D**.
- **Batch Normalisation**.
- **Dropout:** 0.3.

##### Convolutional Block 4

- **Conv2D:** 64 filters, 3×3 kernel.
- **Flatten**.
- **Dropout:** 0.2.

##### Classifier Head

- **Dense:** 64 units, ReLU.
- **Dense:** 2 units, softmax.

#### 2.6.3. Training Procedure

The models are trained for 10 epochs using the AdamW optimizer with the following parameters.

- **Learning rate:** $1\times 10^{-3}$.
- **Weight decay:** 0.004.
- **Batch size:** 128.
- **Loss function:** sparse categorical cross-entropy.

### 2.7. Neural Network Architecture (Vision Transformer)

Vision transformers (ViTs) are a modern machine learning architecture adapted from the transformer model. The emergence of ViTs represents a significant shift in image classification research, introducing a novel alternative to traditional CNNs. Unlike traditional CNNs, which use spatial hierarchies and local receptive fields, ViTs treat images as sequences of patches and learn global relationships through self-attention mechanisms. Originally designed for natural language processing, the transformer architecture relies on self-attention mechanisms to model long-range dependencies within data. ViTs adapt this framework to vision tasks by treating images as sequences of flattened patches, thereby enabling global feature modelling without relying on the inductive biases of convolutions.

**Figure 5:**
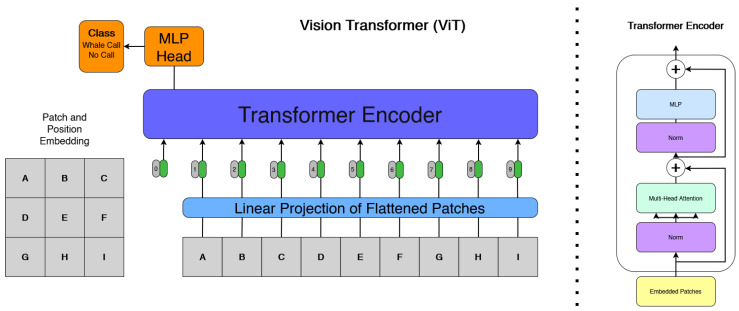
Vision transformer architecture.

It can be seen from the architecture of the ViT in Figure 5 that an image is divided into fixed size patches, each of which is linearly embedded and supplemented with positional information. These embeddings are passed through a series of transformer encoder layers, each comprising multi-head self-attention and feedforward networks. The core idea is to allow the model to learn dependencies and spatial patterns between any parts of the image, regardless of their physical proximity.

According to ref. [33], ViTs offer several theoretical advantages, including flexibility in input size, parallel processing capabilities, and superior modeling of contextual relationships through multi-head self-attention. However, the literature review highlights critical challenges such as high data requirements, computational complexity, slower convergence and limited generalization when trained on small datasets. These limitations contrast sharply with CNNs, which excel in resource constrained scenarios due to their hierarchical feature extraction and strong spatial priors.

Multiple studies compared ViTs and CNNs across diverse domains such as medical imaging, agriculture, and digital holography. Results are mixed: in some cases, ViTs outperformed CNNs, especially when datasets were augmented or pretraining was leveraged. However, in other studies CNNs maintained superior accuracy and robustness, particularly on smaller datasets or noisy inputs. Notably, studies such as ref. [34] show ViTs performing competitively even on relatively small datasets when carefully optimized, suggesting their potential is not strictly limited to large scale benchmarks.

Another relevant contribution shown in ref. [35], formally introduced the ViT architecture and demonstrated its scalability when pretrained on large datasets like ImageNet-21k or JFT-300M. Their results confirm that ViTs can match or exceed CNN performance but only when supplied with vast amounts of labeled data and sufficient training time. This underlines a key conclusion shown across the papers in which ViTs are not inherently superior but become increasingly viable as dataset size and computational resources scale.

Given the scale and structure of the utilized datasets, it is expected that the ViT implementation will not outperform the CNN model in terms of overall accuracy and robustness. Unlike CNNs, which encode strong inductive biases such as locality and translation invariance, ViTs rely almost entirely on the self-attention mechanism to learn spatial relationships, making them more data hungry and less effective on smaller datasets. While the CNN model benefits from these built in priors, allowing it to generalize well with fewer examples, the ViT model starts with a more flexible but unstructured approach, making it prone to overfitting or underfitting unless trained on a sufficiently large and diverse dataset.

If provided with a well annotated industry grade dataset, particularly one that includes a wide variety of spectrogram patterns and real-world acoustic variability, ViT models have demonstrated the ability to match or surpass CNN performance. This scaling property makes ViT an attractive candidate for future applications with access to expansive marine audio datasets. For this study, the ViT model serves not only as a baseline comparison approach but also as part of a future investigation into its applicability for acoustic event detection. It establishes a foundation for further research into transformer-based architectures in marine bioacoustics, where future access to more comprehensive datasets could enable the creation of more accurate and generalizable models than those based on convolutional methods alone.

#### 2.7.1. Implementation

All experiments are conducted with the same datasets utilized by CNN testing. A lightweight ViT architecture is implemented for classification. The model expects inputs of shape [125, 50, 3], where height corresponds to mel/MFCC bins and width to time frames. The first stage converts the 2D image into a sequence of patches without overlapping using a custom patches layer. Each patch is of size 8 × 8 pixels, extracted with stride 8 in both spatial dimensions and VALID padding. Given the input dimensions, this gives 90 patches per image as shown in the equation below.

$$N=\left\lfloor \frac{125}{8}\right\rfloor \times \left\lfloor \frac{50}{8}\right\rfloor =15\times 6=90.$$

Each patch is flattened and treated as a “token” in the subsequent Transformer encoder.

#### 2.7.2. Configuration

- **Input size:** 125×50×3.
- **Patch size:** 8×8.
- **Number of patches:** 90.
- **Patch embedding dimension:** 64.
- **Transformer layers:** 4 encoder blocks.
- **Multi-head attention:** 4 heads, key dimension 64, dropout 0.1.
- **Transformer MLP units:** [128, 64] with GELU activation, dropout 0.1.
- **Classifier head:** Global average pooling followed by a two-layer MLP with units [2048, 1024], GELU activation, dropout 0.3.
- **Output layer:** Dense softmax with two classes (“hump”, “no call”).

#### 2.7.3. Preprocessing Steps

- Pixel normalization to [0, 1] via Rescaling(1/255).
- Per-image standardization.
- Caching and prefetching using AUTOTUNE.

#### 2.7.4. Procedure and Optimizer

- **Learning rate:** $1\times 10^{-3}$ (warm-up + cosine decay schedule).
- **Warm-up duration:** first 2 epochs.
- **Weight decay:** 0.004.
- **Batch size:** 128.
- **Maximum epochs**: 30.

#### 2.7.5. Warm up and Learning Rate Scheduler

- Warm-up duration over first 2 epochs.
- Cosine decay applied for remaining steps.

#### 2.7.6. Training Procedure

- Early stopping (patience = 5, monitor = validation loss).
- Check-pointing to save the best validation loss model.

Delta MFCCs, represent the rate of change in cepstral coefficients over time. This temporal information is crucial for detecting the frequency modulations and rhythmic patterns characteristic of whale calls. Figure 2 shows the delta MFCC of whale calls and no calls. The visualization process is standardized in terms of dimensions and color mapping, resulting in spectrograms that are both visually uniform and information dense.

### 2.8. Dataset Validity, Bias, and Limitations

The constructed dataset provides a practical foundation for evaluating automated detection of humpback whale calls. However, several limitations must be acknowledged to interpret model performance results.

Firstly, the dataset is derived from publicly available online repositories rather than controlled field deployments due to accessibility limitations. These sources widely vary in recording equipment, distance to calls, environmental noise conditions and sampling formats; meanwhile all audio is standardized to a common sample rate and bit depth, and underlying heterogeneity from the acoustic conditions can introduce systematic bias. This includes certain repositories containing clean foreground vocalizations, while others possess high anthropogenic noise levels, causing noise profile distribution imbalance across classes.

Another limitation is presented by asymmetry of the recording durations. The imbalanced audio lengths across the classification categories leads to unequal numbers of generated 6-second windows. Source-based splitting was used to mitigate data leakage but it results in amplification of category size differences where the longer files contribute disproportionately to a single subset. Truncation is applied to extremely long recordings to reduce dominance of some sources, but the process does not eliminate imbalance entirely while also negatively impacting variability in certain acoustic conditions.

Concerns of the manual curation process required to accurately label the humpback and no call tracks are warranted. Although carefully performed, manual cleaning is inherently subjective and can introduce unintentional bias. Segments containing faint or distant calls could be incorrectly be labeled as no-call; and noisy segments could inconsistently be rejected across sources.

Applying augmentation only to the training subset is standard but introduces distributional weaknesses. The model is trained on a mix of natural and augmented samples, while being validated and tested only on natural data. This is methodologically correct for information leakage prevention but the augmented dataset differs in scale and character to the partitions without augmentation. This discrepancy outlines the variability present in the dataset sizes across experiments and needs consideration when comparing model performance across the datasets.

Despite these limitations, the dataset remains valid for evaluation of relative model performance within the scope of this study. The diversity of sources reduces overfitting to a single environment and the source splitting strategy ensures results generalization to unseen audio segments. Future work would benefit from systematically collected field datasets using controlled deployment metadata, balanced class durations to further reduce bias and increase ecological validity.

## 3. Results

The trained models are evaluated using both quantitative performance metrics and visual diagnostics to assess classification effectiveness on the mel spectrogram and MFCC datasets.

### 3.1. Mel Spectrogram

#### 3.1.1. Training and Validation Accuracy and Loss

**Figure 6:**
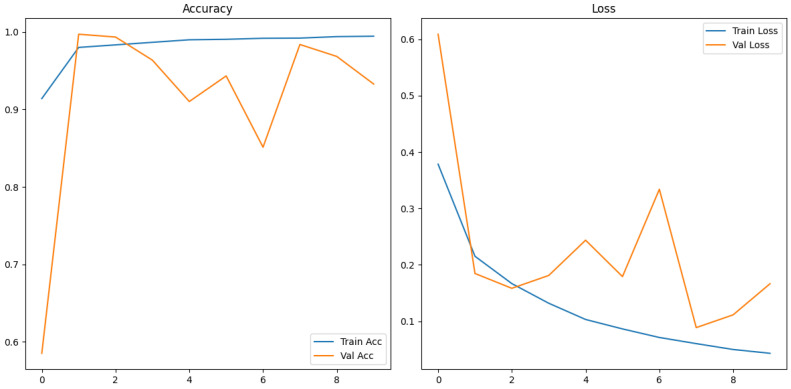
CNN mel spectrogram training and validation.

The training and validation results for the mel spectrogram feature extraction method training with the custom CNN are shown in Figure 6 below.

The evaluation and classification results of the custom CNN trained using mel spectrograms are shown below in Table 3 and Table 4.

Here, the macro average is the mean of all per class F1-scores, the weighted average is also the mean, but for each classes, the number of occurrences weights the per-class F1-score; and support is the number of occurrences.

#### 3.1.2. Confusion Matrix and Classification Results

**Figure 7:**
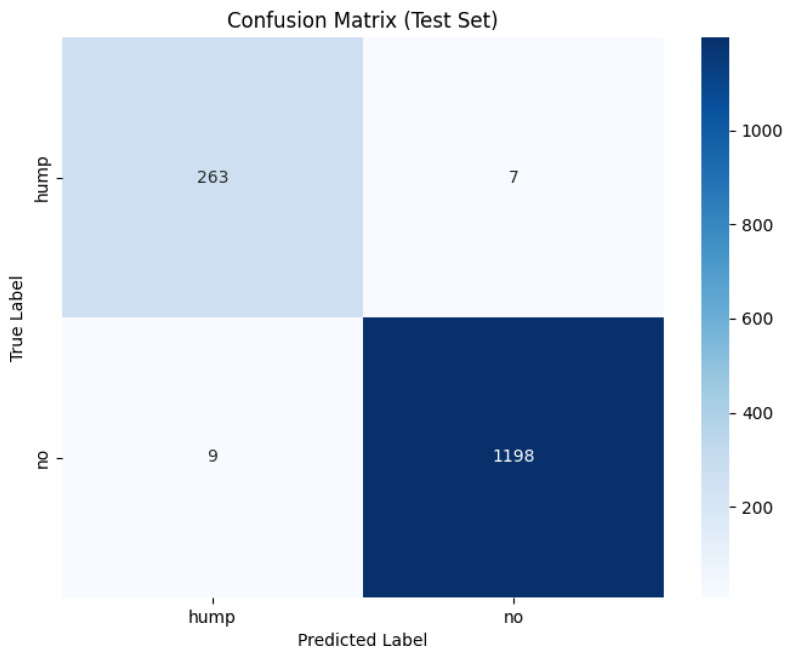
CNN mel spectrogram confusion matrix.

The confusion matrix for the mel spectrogram feature extraction method trained using the custom CNN is shown in Figure 7 below.

#### 3.1.3. ROC and Precision–Recall Curves

**Figure 8:**
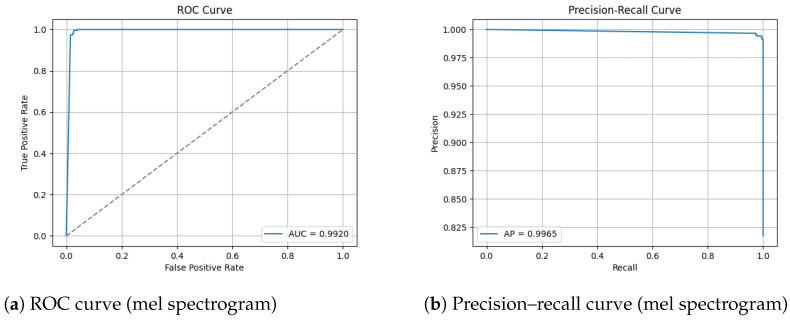
CNN mel spectrogram classification performance visualized through ROC and precision–recall curves. The dashed line represents the line of no discrimination (random classifier, AUC = 0.5).

The ROC and Precision–Recall results for the mel spectrogram feature extraction method trained with the custom CNN are shown in Figure 8 below.

#### 3.1.4. Pretrained Model MobileNetV2

**Figure 9:**
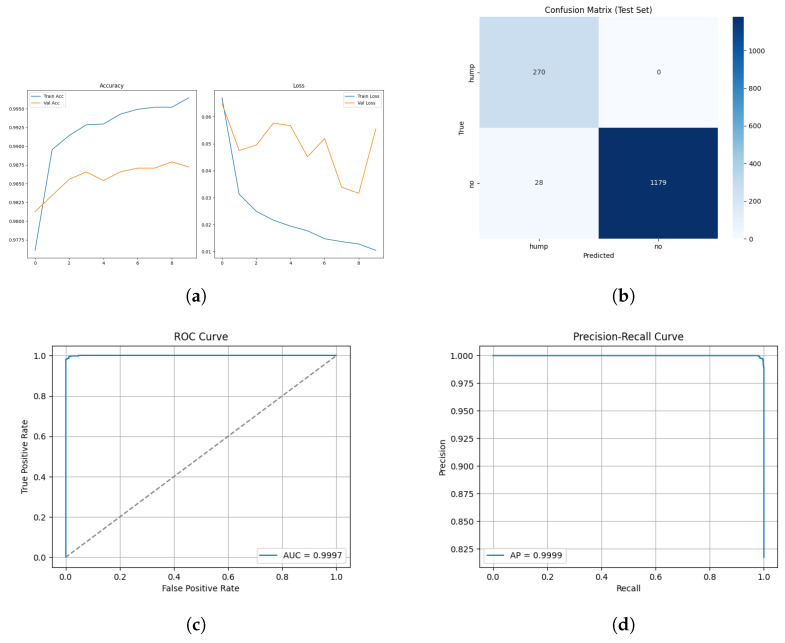
MobileNetV2 classification performance comparison across training behavior, confusion matrix, ROC, and precision–recall characteristics. (**a**) MobileNetV2 mel spectrogram training and validation accuracy and loss; (**b**) MobileNetV2 mel spectrogram confusion matrix; (**c**) MobileNetV2 mel spectrogram ROC curve; (**d**) MobileNetV2 mel spectrogram precision–recall curve.

Figure 9 shows the array of testing metrics obtained using mel spectrogram feature extraction and the MobileNetV2 CNN.

The evaluation and classification results of the MobileNetV2 CNN trained using mel spectrograms are shown below in Table 5 and Table 6.

#### 3.1.5. No Augmentation

**Figure 10:**
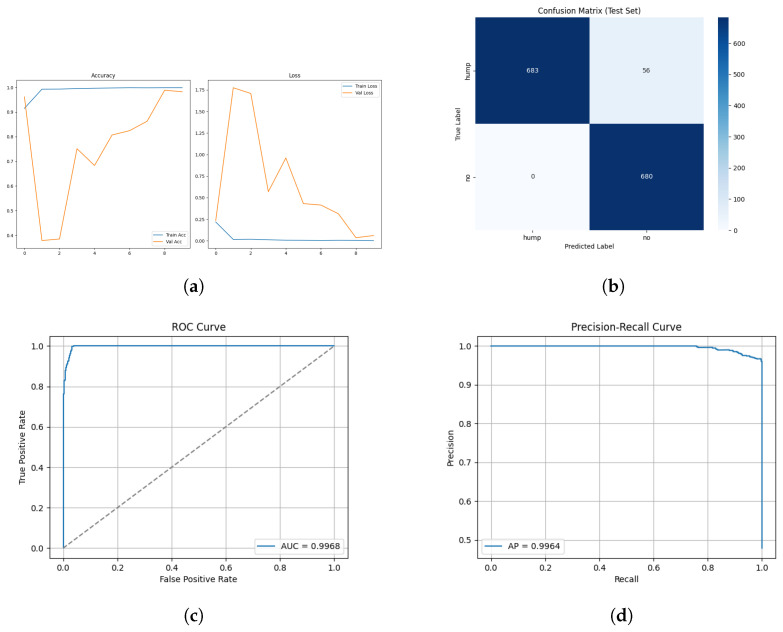
CNN classification performance comparison across training behavior, confusion matrix, ROC, and precision–recall characteristics. (**a**) CNN mel spectrogram training and validation accuracy and loss (no augmentation); (**b**) CNN mel spectrogram confusion matrix (no augmentation); (**c**) CNN mel spectrogram ROC curve (no augmentation); (**d**) CNN mel spectrogram precision–recall curve (no augmentation).

**Figure 11:**
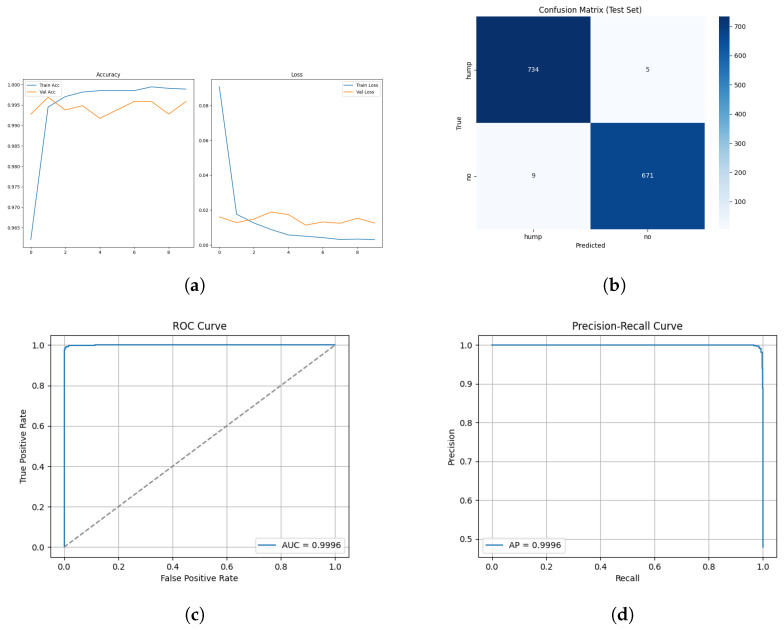
MobileNetV2 classification performance comparison across training behavior, confusion matrix, ROC, and precision–recall characteristics. (**a**) MobileNetV2 mel spectrogram training and validation accuracy and loss (no augmentation); (**b**) MobileNetV2 mel spectrogram confusion matrix (no augmentation); (**c**) MobileNetV2 mel spectrogram ROC curve (no augmentation); (**d**) MobileNetV2 mel spectrogram precision–recall curve (no augmentation).

Figure 10 and Figure 11 show the array of testing metrics obtained using mel spectrogram feature extraction and the MobileNetV2 CNN without augmentation.

The evaluation and classification results of the custom CNN trained using mel spectrograms without augmentation are shown below in Table 7 and Table 8.

The evaluation and classification results of the MobileNetV2 CNN trained using mel spectrograms without augmentation are shown below in Table 9 and Table 10.

#### 3.1.6. Discussion

The custom CNN models trained on mel spectrogram representations demonstrated consistently strong performance across augmented and non-augmented datasets, though notably different learning dynamics and generalization characteristics are present.

The model applied to the augmented dataset achieved the strongest performance, 98.92% accuracy, 0.96 MCC and low error rates. The confusion matrix shows minimal misclassifications for both classes, correctly identifying 263/270 humpbacks and 1198/1207 no calls from the unseen test set. Despite oscillations present in validation loss, the ROC and precision-recall curves indicate a robust decision boundary where minimal degradation appears across recall thresholds. The performance improvement from augmentation demonstrates the techniques effectively increased the representational space, allowing the CNN to learn invariance which boosts robustness and generalization in diverse acoustic conditions. The non-augmented dataset produced strong but reduced performance overall. In conclusion, CNN trained on mel spectrograms demonstrates suitability for small windows of humpback detection and we can conclude in this test scenario that augmentation improves generalization and mitigates dataset bias when applied to mel spectrogram feature representations.

The mobileNetV2 architecture demonstrated consistently accurate performance on the augmented and non-augmented mel spectrogram datasets. ImageNet’s initialized weights provided substantial ability for the model to converge rapidly to achieve high accuracy. Augmented dataset reached 98.1% test accuracy and an MCC of 0.94, demonstrating the pretrained models capability of generalizing across the range of augmented acoustic conditions provided. It achieved a false positive rate of 0 and excellent recall for humpback calls with a false negative rate of 2.3%. The exhibited fluctuating validation accuracy could suggest the model is partially reliant on features inherited from ImageNet, which may not align with the structure of bioacoustic spectrograms. Notably, training the architecture without augmentation boosted accuracy, achieving 99% test accuracy and an MCC of 0.98. As acoustic conditions of this dataset are more homogeneous, it is possible the architecture is overfitting to stylistic artifacts of the spectrogram rather than learning species specific call characteristics. It is possible that MobileNetV2 can outperform the custom architectures; however, further testing with new exhaustive datasets would be required for a conclusion to be drawn.

### 3.2. MFCC

#### 3.2.1. Training and Validation Accuracy and Loss

**Figure 12:**
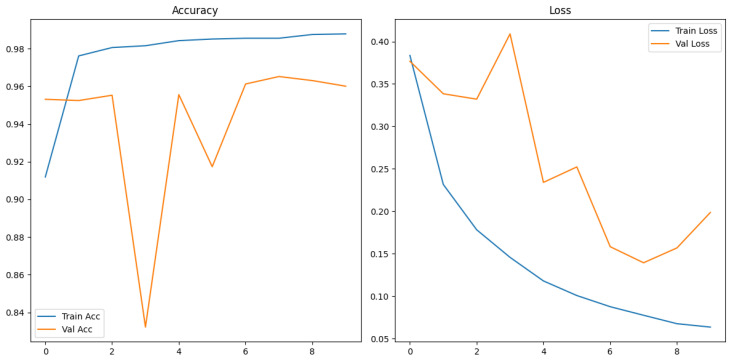
CNN MFCC training and validation.

Figure 12 shows the training and validation graphs using MFCC feature extraction and the custom CNN.

The evaluation and classification results of the custom CNN trained using MFCC extraction methods are shown below in Table 11 and Table 12.

#### 3.2.2. Confusion Matrix and Classification Results

**Figure 13:**
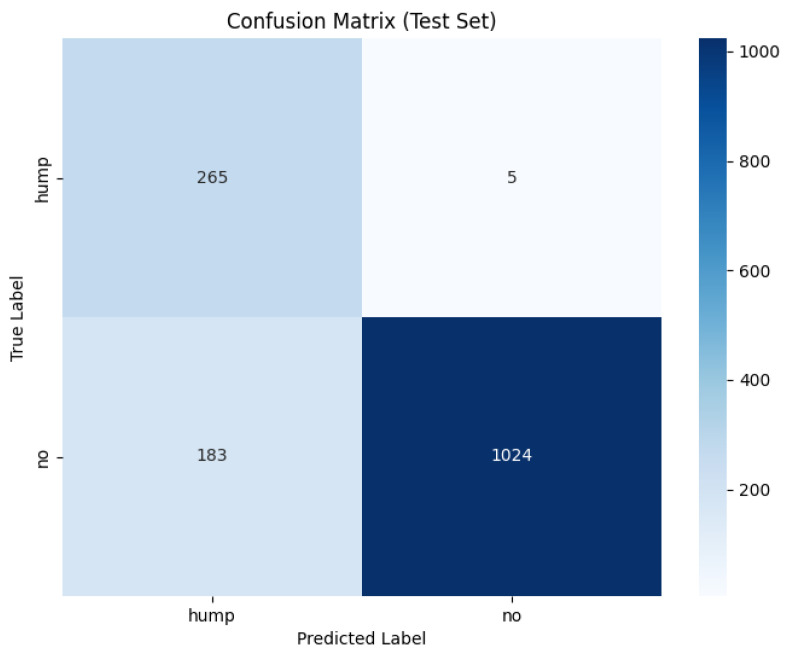
CNN MFCC confusion matrix.

The confusion matrix for the MFCC feature extraction method trained using the custom CNN is shown in Figure 13 below.

#### 3.2.3. ROC and Precision–Recall Curves

**Figure 14:**
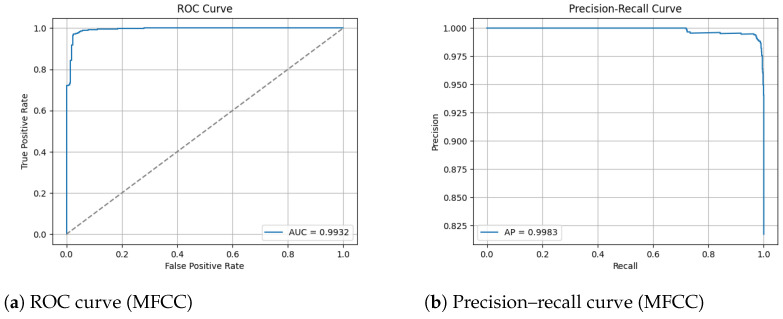
CNN MFCC classification performance visualized through ROC and precision–recall curves.

The ROC and Precision–Recall results for the MFCC feature extraction method trained with the custom CNN are shown in Figure 14 below. Note that the dashed line represents the line of no discrimination (random classifier, AUC = 0.5).

#### 3.2.4. Pretrained Model MobileNetV2

**Figure 15:**
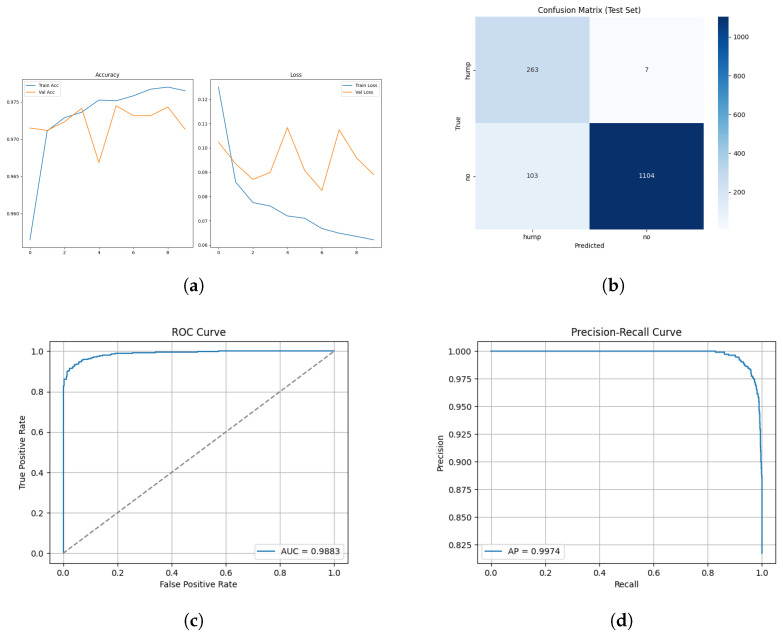
MobileNetV2 MFCC classification performance comparison across training behavior, confusion matrix, ROC, and precision–recall characteristics. (**a**) MobileNetV2 MFCC training and validation accuracy and loss; (**b**) MobileNetV2 MFCC confusion matrix; (**c**) MobileNetV2 MFCC ROC curve; (**d**) MobileNetV2 MFCC precision–recall curve.

Figure 15 shows the array of testing metrics obtained using MFCC feature extraction and the MobileNetV2 CNN.

The evaluation and classification results of the MobileNetV2 CNN trained using MFCC extraction methods are shown below in Table 13 and Table 14.

#### 3.2.5. No Augmentation

**Figure 16:**
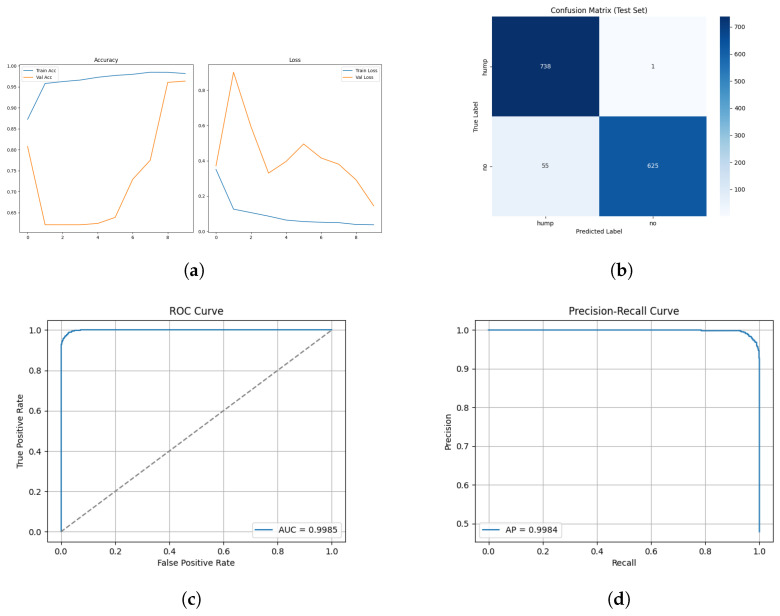
CNN MFCC classification performance comparison across training behavior, confusion matrix, ROC, and precision–recall characteristics. (**a**) CNN MFCC training and validation accuracy and loss (no augmentation); (**b**) CNN MFCC confusion matrix (no augmentation); (**c**) CNN MFCC ROC curve (no augmentation); (**d**) CNN MFCC precision–recall curve (no augmentation).

**Figure 17:**
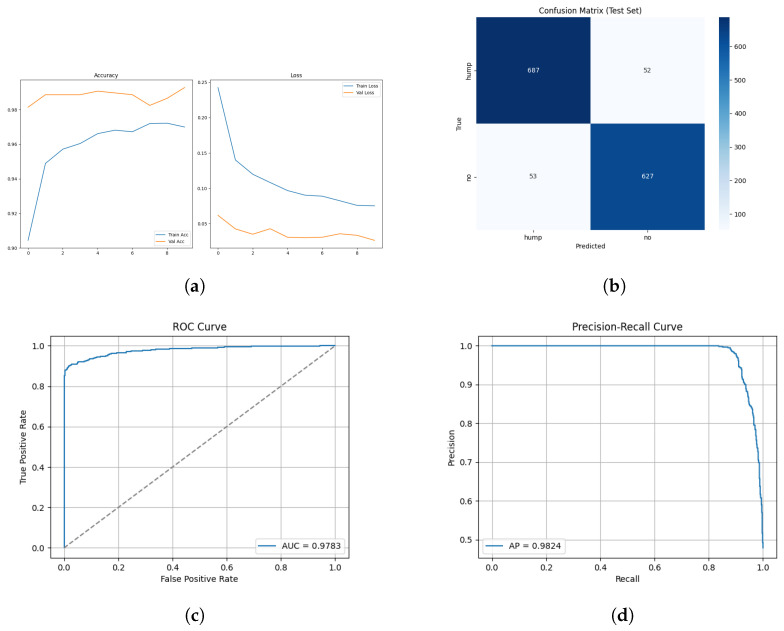
MobileNetV2 classification performance comparison across training behavior, confusion matrix, ROC, and precision–recall characteristics. (**a**) MobileNetV2 MFCC training and validation accuracy and loss (no augmentation); (**b**) MobileNetV2 MFCC confusion matrix (no augmentation); (**c**) MobileNetV2 MFCC ROC curve (no augmentation); (**d**) MobileNetV2 MFCC precision–recall curve (no augmentation).

Figure 16 and Figure 17 show the array of testing metrics obtained using MFCC feature extraction and the MobileNetV2 CNN without augmentation.

The evaluation and classification results of the custom CNN trained using MFCC extraction methods without augmentation are shown below in Table 15 and Table 16.

The evaluation and classification results of the MobileNetV2 CNN trained using MFCC extraction methods without augmentation are shown below in Table 17 and Table 18.

#### 3.2.6. Discussion

The custom CNN models trained on MFCC representation show different behaviour to the effects of augmentation.

The augmented MFCC model reached high validation accuracy (95%) throughout training yet the final test diminished substantially to 87% with a poor false negative rate of 15%, indicating a severe generalization failure. Examining the confusion matrix displays the failure of frequent misclassifications in positive humpback calls. This behaviour suggests the augmentations applied are poorly aligned with MFCC representations where the cepstral coefficients have been altered disproportionately in comparison to mel spectrogram characteristics. The augmentation method has potentially introduced distortions that push MFCC samples beyond the manifold of realistic whale calls, causing an overfit to the augmentation artifacts as opposed to the biological structure.

The dataset without augmentation substantially increased performance of the custom CNN, achieving test accuracy of 96%, false negative rate of 8% and a high 0.92 MCC. The confusion matrix shows humpback calls are predicted with high accuracy (738/739), but a no-call is a acenario occasionally misclassified as a humpback call with 55 false positives. The testing demonstrates MFCCs retain information to reliably distinguish calls, provided clean data and without adverse augmentation distorting feature geometry. The testing in this study demonstrates several important insights: MFCCs are sensitive to augmentation; MFCCs generalize less reliably than mel spectrograms or that poor augmentation methods for the MFCC feature representation may have been utilized.

The performance of MobileNetV2 applied to MFCC representations further presents evidence that CNN architectures do not generalize as effectively to this feature domain as well as mel spectrograms. Despite strong validation during training, the augmentation and non-augmentation configurations exhibit lower test accuracy of 92–93% when compared with mel spectrograms 98–99%. In both augmentation configurations, MobileNetV2 achieves high precision for the no call class (91–99%), presenting a reliable ability to reject signals without vocalization. Low false positive rates (1.8–7%), indicate that no-whale-call nioses are rarely identified as whale calls. The mediocre MCC values (0.79–0.85) demonstrate average classification reliability across classes. Its weaknesses are concentrated of misclassifications within the humpback call class. Data augmentation for MFCC again presents harm to MobileNetV2’s ability to generalize, suppressing whale call recall. This suggests that augmentation–or this specific augmentation–distorts the cepstral coefficients detrimentally. It is plausible the pretrained feature extractor does not align with the MFCC topology, as ImageNet has trained MobileNetV2 to learn hierarchical spatial filters for natural images. Comparing this to MFCCs method of reducing feature domain for optimization purposes, the different goals do not combine suitably.

### 3.3. Vision Transformer

#### 3.3.1. ViT Mel Spectrogram

**Figure 18:**
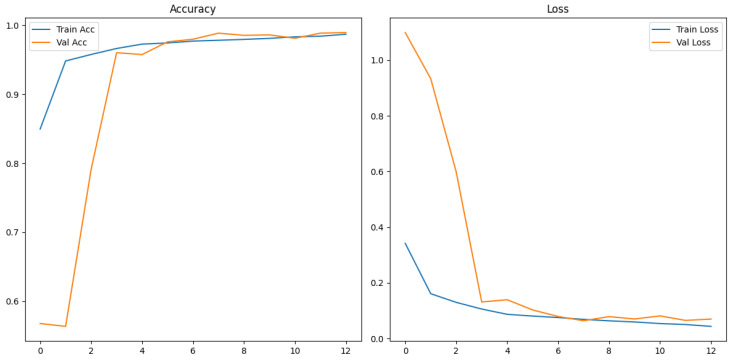
Vision transformer mel apectrogram training and validation.

**Figure 19:**
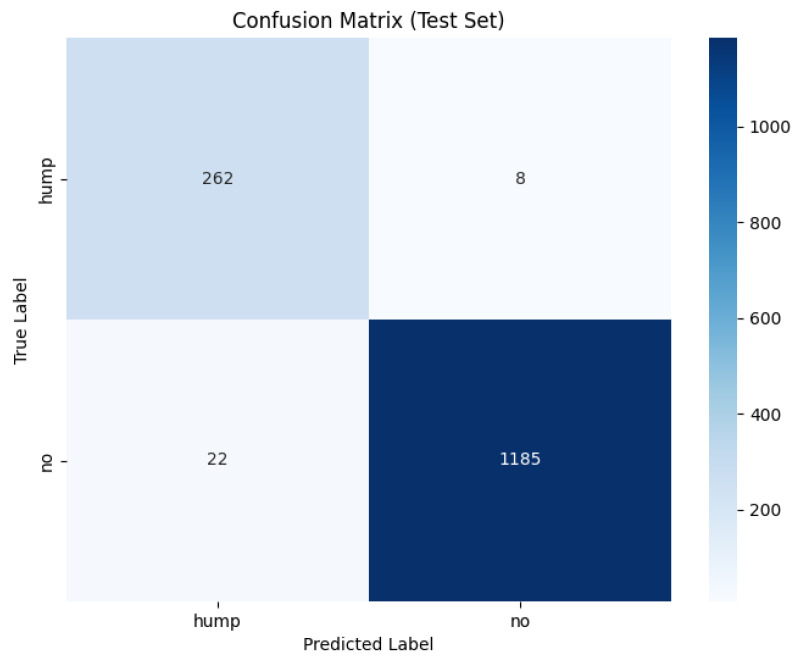
ViT mel spectrogram confusion matrix.

The training and validation results for the mel spectrogram feature extraction method training with the custom ViT are shown in Figure 18 and Figure 19 below.

The evaluation and classification results of the Vision Transformer trained using mel spectrograms are shown below in Table 19 and Table 20.

#### 3.3.2. ROC and Precision–Recall Curves

**Figure 20:**
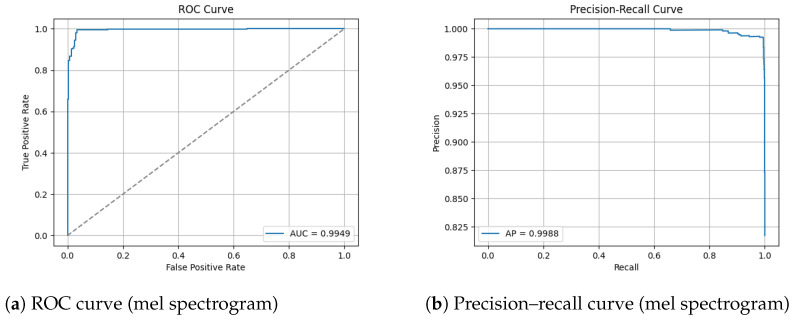
ViT mel spectrogram classification performance visualized through ROC and Precision–Recall curves.

The ROC and Precision–Recall results for the mel spectrogram feature extraction method training with the custom ViT are shown in Figure 20 below.

#### 3.3.3. No Augmentation

**Figure 21:**
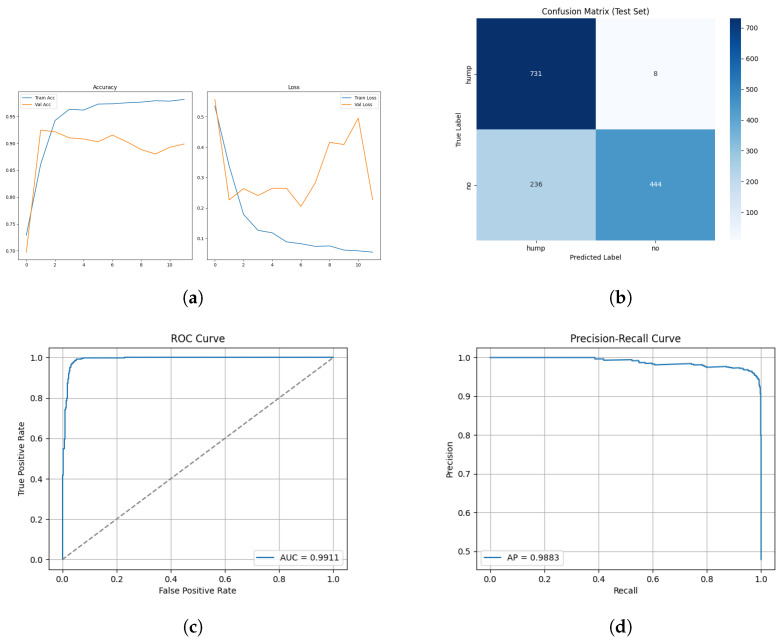
ViT classification performance comparison across training behavior, confusion matrix, ROC, and precision–recall characteristics. (**a**) ViT spectrogram training and validation accuracy and loss (no augmentation); (**b**) ViT spectrogram confusion matrix (no augmentation); (**c**) ViT spectrogram ROC curve (no augmentation); (**d**) ViT spectrogram precision–recall curve (no augmentation).

Figure 21 shows the array of testing metrics obtained using mel spectrogram feature extraction and the custom ViT without augmentation.

The evaluation and classification results of the Vision Transformer trained using mel spectrograms without augmentation are shown below in Table 21 and Table 22.

#### 3.3.4. ViT MFCC

**Figure 22:**
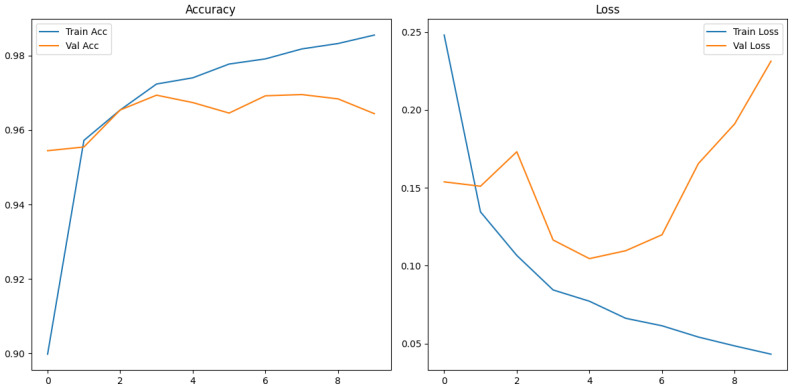
ViT MFCC training and validation Curves.

**Figure 23:**
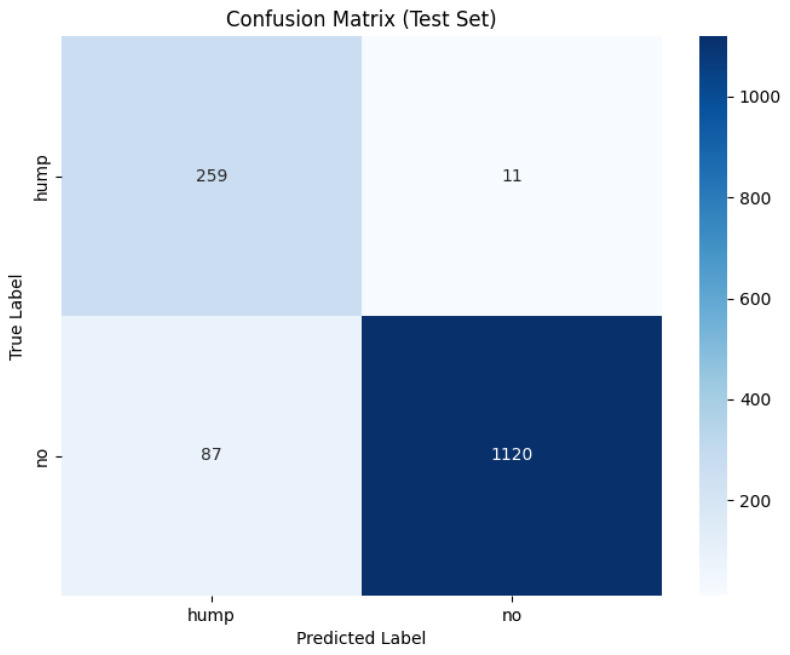
ViT MFCC confusion matrix.

Figure 22 and Figure 23 show the training and validation graphs using MFCC feature extraction and the custom ViT.

The evaluation and classification results of the Vision Transformer trained using MFCC extraction methods are shown below in Table 23 and Table 24.

#### 3.3.5. ROC and Precision–Recall Curves

**Figure 24:**
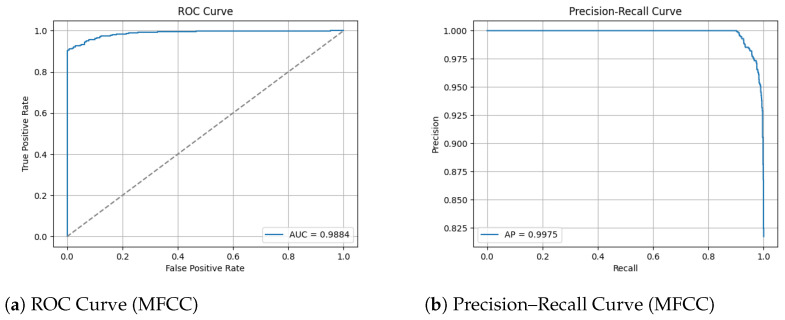
ViT MFCC classification performance visualized through ROC and Precision–Recall curves.

The ROC and Precision–Recall results for the mel spectrogram feature extraction method training with the custom ViT are shown in Figure 24 below.

#### 3.3.6. No Augmentation

**Figure 25:**
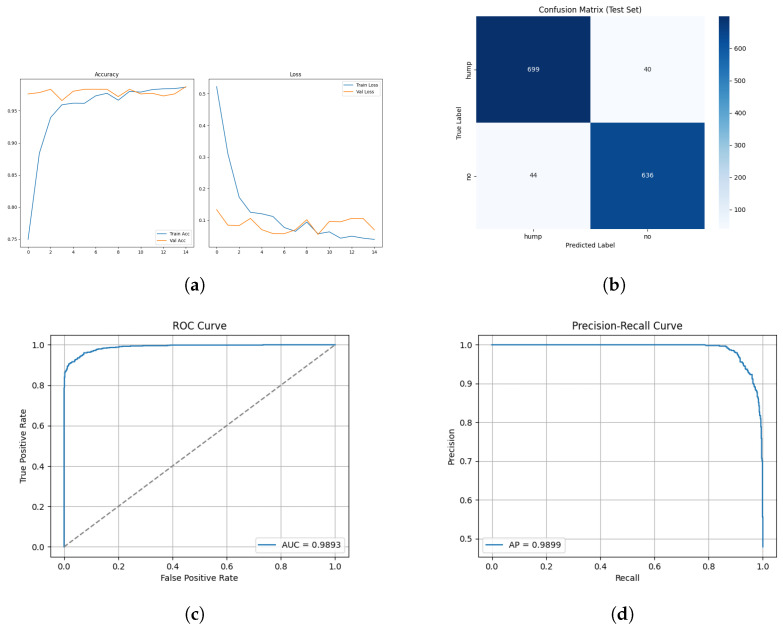
ViT MFCC classification performance comparison across training behavior, confusion matrix, ROC, and precision–recall characteristics. (**a**) ViT MFCC training and validation accuracy and loss (no augmentation); (**b**) ViT MFCC confusion matrix (no augmentation); (**c**) ViT MFCC ROC curve (no augmentation); (**d**) ViT MFCC precision–recall curve (no augmentation).

Figure 25 shows the array of testing metrics obtained using mel spectrogram feature extraction and the custom Vit without augmentation.

The evaluation and classification results of the Vision Transformer trained using MFCC extraction methods without augmentation are shown below in Table 25 and Table 26.

#### 3.3.7. Discussion

The ViTs exhibited mixed performance with mel spectrogram representations, demonstrating a strong classification ability with data-augmented sets and notable instability with non-augmented sets, and highlighting the sensitivity of transformer models to dataset scale, diversity and regularization. On the augmented dataset, ViT achieved 98% test accuracy and an MCC of 0.93, indicating strong classification performance. The confusion matrix reveals a reasonable 2% false negative rate and a slightly higher false positive rate of 3%. The non-augmented dataset performed substantially worse, with 83% test accuracy and a poor MCC score of 0.69, the lowest of all tested models. The confusion matrix highlights a heavily imbalanced error behaviour, it correctly identified 731/739 humpback vocalizations but misclassified a large proportion of no-call samples, resulting in recall of the no class to drop to 65%. This suggests that the non-augmented dataset is of insufficient size for transformer learning. The ViT is unable to learn meaningful relationships between spectrogram patches. It shows that it is heavily overfitted to humpback call features, the model’s over prediction of humpback calls infers a poor generalization capability.

The ViT trained using MFCC achieved a test accuracy of 93%, with a notably low precision for humpback calls indicating substantial false positives at 4%. It achieved a low MCC score of 0.81. This shows that the model often mistakes no-call noises as a humback whale calls. The performance of ViT on MFCC without augmentation again shows result of augmentation negatively impacting that MFCC representational structure due to an improved MCC value of 0.88. This raises questions about the performance of ViT on an MFCC dataset of suitable size without requiring augmentation. The results imply that ViT architectures are viable for acoustic spectrogram classification when extensive augmentation or large annotated datasets are available. CNN models remain computationally efficient, stable and less data dependent.

MobileNetV2 exhibits the highest accuracy of all tests on the non-augmented dataset. The custom CNN architectures however demonstrate higher humpback recall, better overall accuracy, superior class balance robustness, and greater stability under augmentation.

### 3.4. Existing Methods

Automated whale call detection has evolved substantially over the past two decades, driven by increasing dataset scale, environmental variability and the need for reliable long-term passive acoustic monitoring. Early approaches relied predominantly on handcrafted acoustic features combined with classical classifiers, whereas more recent studies have shifted toward deep learning architectures operating directly on time–frequency representations. Performance differences across studies are strongly influenced by task formulation, particularly analysis window duration, feature representation and classification objective.

#### 3.4.1. Classical Feature-Based Approaches

Early whale call detection systems primarily employed handcrafted features such as MFCCs, wavelet coefficients and energy-based spectral descriptors, coupled with classifiers including linear discriminant analysis, support vector machines, principal component analysis and dictionary learning [36,37]. These methods demonstrated reasonable performances under controlled conditions and limited acoustic variability but were often sensitive to background noise, call variability and changes in recording environments [36,37]. As PAM datasets expanded in both duration and geographic diversity, these approaches showed limited robustness and scalability due to the substantial manual effort required for feature engineering and tuning as well as the degradation of performance in complex underwater environments [36,37]. These limitations motivated the transition toward more robust and scalable data-driven learning methods.

#### 3.4.2. Deep Learning and Long Window Detection

The adoption of CNNs marked a significant methodological shift by enabling end to end learning of discriminative time–frequency features directly from spectrogram representations [38,39]. This approach overcomes the constraints of manual feature engineering, allowing models to learn task-specific representations from raw or minimally processed acoustic data, improving generalization across diverse acoustic environments and enhancing robustness to noise [38,39]. A representative example is the binary humpback whale classification system proposed by [38], which employs mel spectrogram inputs and a modified ResNet 50 architecture operating on 75-s audio windows. This study was evaluated on an exceptionally large PAM dataset comprising approximately 187,000 h of recordings collected over 14 years from 13 sites using bottom-mounted High-Frequency Acoustic Recording Packages. A key contribution of this work is the use of active learning to iteratively expand the labelled dataset, ultimately producing 292 hours of expert-annotated training data. By prioritizing high-confidence false positives and ambiguous detections for re-annotation, the authors generated hard negative examples that substantially improved generalization across diverse acoustic environments [38]. The resulting system achieved an AUC-ROC of 0.992, demonstrating a strong performance at large scale. However, the use of 75-s analysis windows fundamentally frames this task as long-duration song detection rather than short-window call presence detection. The extended temporal context allows the model to utilize harmonic continuity and song structure, substantially reducing ambiguity under low signal-to-noise conditions. Consequently, although this approach achieves an excellent performance, its methodological assumptions and task formulation differ markedly from those of short-window detection problems and direct performance comparisons must be interpreted with caution.

#### 3.4.3. Short-Window and Multi-Class CNN-Based Approaches

In contrast to long-window song detection, other studies focus on shorter segmented call samples and more specific classification objectives. The study by [40] proposes a Multi-Scale Deep Feature Aggregation CNN for the multi-class regional classification of humpback whale calls using MFCC features. Their dataset consists of 400 one-hour hydrophone recordings segmented into call containing samples, which were expanded to approximately 23 h of data through augmentation techniques including time stretching, pitch shifting and noise addition [40]. The proposed model achieved a 95% classification accuracy with an AUC-ROC of 0.98 across four marine regions. While these results highlight the effectiveness of multi-scale CNN architectures, the task formulation differs notably from short-window binary detection. Classification is performed on segmented call samples rather than fixed-length windows and the objective is regional classification rather than call presence detection. These differences have important implications for feature suitability and for interpreting reported performance. [40] assumes that input segments contain calls and focuses on discriminating among classes, whereas short-window detectors must also distinguish whale calls from diverse non-whale background events.

#### 3.4.4. Feature Representation and Window Length Effects

The discrepancy between the strong MFCC performance reported by [40] and the comparatively weaker MFCC results observed in the present study can be attributed primarily to differences in temporal context and task definition. MFCCs are effective at capturing the averaged spectral envelope over longer or acoustically stable segments, making them well suited to segmented call classification and multi-class tasks [40]. However, in short fixed-length windows such as 6 s in the present study, MFCC compression discards the fine-grained harmonic structure and transient information that are critical for detecting partial or weak calls. This leads to increased false negative rates and reduced sensitivity under constrained temporal context. As window duration decreases, the detection task becomes fundamentally more challenging, shifting from recognizing sustained vocal patterns to identifying fragmented harmonics with limited contextual information. Under these conditions, feature representations that preserve detailed time–frequency structure such as mel spectrograms become increasingly advantageous. This trend helps explain the consistently superior performance of mel spectrogram-based models observed in the present study relative to MFCC-based approaches.

#### 3.4.5. Emerging Architectures and Alternative Representations

Recent research has further extended deep learning approaches through the development of hybrid and multi-scale architectures. The study by [41] introduces a Convolutional Vision Transformer (CvT) model for North Atlantic right whale up call detection using spectrogram image representations. By combining convolutional inductive biases with transformer-based attention mechanisms, this approach captures both local spectral patterns and broader contextual relationships [41]. Their model achieved an approximately 97.25% accuracy and a high F1- score while demonstrating an improved parameter efficiency relative to standard Vision Transformers [41]. This work reflects a broader trend toward architectures that balance representational power with practical inference efficiency. Complementary work has explored alternative time–frequency representations beyond conventional mel spectrograms. For instance, ref. [42] investigated whale call detection using wavelet-based scalograms paired with Hidden Markov Model classification for Antarctic blue whale sounds. Wavelet transforms provide multi-resolution analysis that preserves transient signal structure across multiple temporal scales, offering excellent time–frequency localization and making them well suited to short non-stationary vocalizations and intricate cetacean call patterns [42]. While such approaches demonstrate a competitive detection performance particularly under low-signal-to-noise conditions, their increased computational cost and higher feature dimensionality typically limit real-time applicability compared with mel spectrogram pipelines [42].

#### 3.4.6. Overall Analysis

Taken together, existing methods reveal a clear progression from classical handcrafted feature pipelines toward deep learning systems operating on rich time–frequency representations. Early approaches addressed basic detection under constrained conditions but lacked robustness and scalability. CNN-based spectrogram classifiers significantly improved generalization by learning hierarchical acoustic features, while recent multi-scale and hybrid architectures further enhance performance by integrating information across temporal and spectral resolutions.

Window duration and task formulation play a defining role in determining both achievable performance and feature suitability. Long-window approaches benefit from extended temporal context and are well suited to song detection, whereas short-window detection imposes stricter requirements on feature fidelity and temporal resolution. In this context, mel spectrogram representations offer a favourable balance between representational richness and computational efficiency, explaining their superior performance in short-window detection tasks relative to MFCCs. These observations underscore the importance of evaluating detection methods within the context of their intended application rather than relying solely on isolated performance metrics. Precision, recall, F1-score and AUC-ROC remain vital for assessing model efficacy but must be interpreted with respect to window length, signal-to-noise regime and operational constraints [36,39].

To further contextualize the performance of the architectures and feature representations studied, the results were compared alongside representative studies of whale classification and detection. A summary of this comparison is included in the Results Comparison section in Table 29.

### 3.5. Results Comparison

A comparison of the models across all feature extraction methods and with or without augmentation is shown in Table 27 below.

A comparison of false positive and false negative rates across all testing scenarios are shown below in Table 28.

A comparison between the highest performing test scenarios and existing methods is shown in Table 29 below.

Across all experiments, model performance was strongly influenced by the three main testing approaches of feature representation, network architecture and presence or absence of data augmentation. The interaction between these factors produced consistent, interpretable patterns, highlighting the strengths and limitations of the modeling approaches for humpback whale call detection.

A clear trend across all architectures is the higher performance of mel spectrograms over MFCC features. Models trained on mel spectrogram inputs consistently achieved the highest accuracy, MCCs and balanced precision recall performance. This suggests that the advantage of mel spectrograms in preserving high-resolution time–frequency structure is directly linked to classifying the harmonic nature of whale vocalizations.

MFCCs generally produced lower MCC scores (0.70–0.88), had higher false negative rates, particular for ViT without augmentation (FNR = 34.7%) and lower whale call precision, particularly under augmentation (59% for CNN MFCC with augmentation). While MFCCs can still yield competitive accuracy under some conditions, overall the performance was consistently lower than mel spectrograms. Further testing of ViT on a larger MFCC dataset without augmentation would provide beneficial results to enhance this study.

Among all models, MobileNetV2 consistently achieved the highest and most stable performance. It achieved the highest results with mel spectrogram on the non-augmented dataset, this being 99.01% test accuracy, 99% precision and recall and an MCC of 0.98. The model clearly benefits from transfer learning, leveraging feature extraction layers pretrained on ImageNet that provided strong potential for 2D pattern recognition. Its robustness is further evidenced by its capability of maintaining performance on MFCC experiments, where other architectures would notably degrade. The results were weaker (92–93% test accuracy) but still outperformed the other models. MobileNetV2 exhibits the highest accuracy of all tests on the non-augmented dataset. The custom CNN architectures however demonstrate higher humpback recall, better overall accuracy, superior class-balance robustness and greater stability under augmentation. Further testing is required to stress the generalization capabilities of the models.

The comparative results presented in Table 29 situate the proposed models within a broad range of whale detection and classification approaches spanning benchmark evaluations, classical feature-based pipelines, CNN-based detectors and emerging hybrid architectures. Across these studies, reported performance varies substantially, reflecting differences in classification objective, temporal context and feature representation rather than architectural complexity alone. Consequently, performance metrics such as accuracy, F1-score and AUC-ROC must be interpreted in relation to the specific detection or classification scenario being addressed.

Benchmark and multi-class studies illustrate that performance generally decreases as task complexity increases. Generalized benchmark evaluations and multi-species or multi-class classification problems typically report lower or more variable F1-scores than binary detection tasks, highlighting the added difficulty of discriminating across multiple call types, species and acoustic conditions [37,39]. While these studies provide valuable context, they are not directly comparable to short-window call presence detection, where the objective is limited to identifying whether a call occurs within a brief temporal segment rather than performing careful classification.

Classical approaches based on handcrafted features exhibit a strong dependence on signal segmentation strategy and acoustic context. Wavelet-based binary detection methods operating under short-window conditions typically report F1-scores below 90%, reflecting sensitivity to background noise and reduced robustness when calls are partial or weak [42]. In contrast, MFCC-based systems can achieve substantially higher performances when applied to structured classification tasks using pre-segmented call-level audio. For instance, ref. [40] reports both accuracy and F1-scores of approximately 95% for multi-class regional classification, indicating that MFCCs are well suited to tasks involving longer, acoustically stable segments but may become limiting under short-window detection scenarios.

CNN-based methods operating on richer time–frequency representations such as mel spectrograms or PCEN consistently report strong performance across both binary and multi-class tasks. Recent studies commonly achieve F1-scores in the range of 95–97%, with AUC-ROC values approaching or exceeding 0.99 [38,41]. However, many of these systems rely on substantially longer analysis windows or pre-segmented inputs, enabling models to exploit extended temporal context, harmonic continuity and song structure. This fundamentally simplifies the detection problem relative to short fixed-window detection, where models must identify partial calls embedded within arbitrary and often noisy acoustic backgrounds.

Within modern methodologies, the proposed Custom CNN and MobileNetV2 models demonstrate that a competitive detection performance can be achieved under short window constraints. By operating on fixed 6-second analysis windows and using mel spectrogram representations, these models address a detection scenario representative of continuous passive acoustic monitoring. Their reported accuracy, F1-score and AUC-ROC values fall within the range observed for recent CNN-based systems, despite differences in dataset scale, temporal context and task formulation. This consistency highlights the effectiveness of mel spectrogram representations in preserving critical time–frequency structures under limited contextual information.

Overall, the synthesis of the results in Table 29 indicates that whale detection and classification performance is jointly shaped by analysis window duration, input representation and classification objective. Long-window and segmented input approaches benefit from increased temporal context, while short-window detection imposes stricter requirements on representation fidelity and robustness. These observations reinforce the importance of evaluating detection methods within their intended operational context and interpreting reported performance metrics accordingly, rather than in isolation.

### 3.6. AODN Dataset Discoveries

The AODN dataset represents a significant manual undertaking, even after the model has been used to categorize selected segments of the audio. The majority of the audio is correctly classified as “no call”, which aligns with expectations, as whale calls would typically constitute only a small fraction of continuous marine recordings. There is also no guarantee that whale calls are present in the segments that have been tested on. Encouragingly, the model is not misclassifying most miscellaneous marine animal sounds such as squeaks and honking noises as whale calls, suggesting that it has learned to distinguish between relevant and irrelevant acoustic features. However, the model does exhibit some classification errors with specific types of low-frequency noise. It particularly tends to incorrectly label certain lower frequency sounds produced by large vessels, as well as piercingly loud rising tones that appear to be the hydrophone experiencing feedback issues. These false positives may result from the spectral similarity between these tonal events and actual whale calls, especially in the low frequency range. These edge cases highlight an opportunity for further refinement in both preprocessing and model sensitivity to unusual but irrelevant low frequency patterns. It is unsurprising that certain sounds are misinterpreted, as some audio present in this dataset has not been fed to the current model which showcases the generalization capabilities of the models.

## 4. Future Work

### 4.1. Model Improvement

One avenue for improvement is the implementation and evaluation of a hybrid model architecture that combines CNNs with ViTs. While CNNs excel at learning local spatial features through hierarchical convolutional layers, ViTs are adept at capturing long-range dependencies and global context via self attentive mechanisms. A hybrid configuration could allow early CNN layers to extract fundamental spatial patterns, followed by ViT layers that model more abstract relationships across patches. This combination is likely to yield better performance in acoustic classification tasks that involve complex or overlapping temporal structures. In addition to architectural combination, future work should explore more advanced models specifically tailored for multi-class classification. Unlike the current binary implementation, multi-class systems would need to discriminate between multiple marine species, background noises and anthropogenic sources. This may involve deeper networks, class specific attention mechanisms or even the use of multi label output heads if sound events overlap. It may also require modifications to the loss function (e.g., focal loss or label smoothing) and careful management of class imbalance during training. Furthermore, including a pretrained ViT model would provide a more comprehensive comparative analysis.

### 4.2. Dataset Improvement

The dataset could be further expanded by incorporating additional field sourced whale call recordings, as well as acoustic samples from a wider range of marine species, including dolphins, seals, and fish with distinctive vocalizations. To enhance the robustness of the classifier and its applicability in real world ocean monitoring scenarios, the dataset could also include categories for non-biological acoustic events, such as vessel noise (e.g., ship engines, propeller cavitation), seismic activity, underwater construction and ambient ocean sounds like wave turbulence or rainfall. This broader scope would not only improve the model’s ability to distinguish between biological and anthropogenic sources but also contribute to applications such as marine traffic monitoring, habitat health assessment, and the detection of underwater noise pollution.

### 4.3. Signal Localization

To further expand upon the concept presented, the ability to combine the machine learning detection capability with a system of transient detectors and a subsequent localization algorithm introduces the potential for a comprehensive passive acoustic monitoring framework. Such a system would not only identify the presence of marine mammals in real time but also triangulate their approximate positions using arrays of detector–sensors. This integration would allow researchers to track movement patterns, estimate population distributions and even identify behavioural hot spots over time. When deployed over extended periods, this approach could form the foundation of a scalable, automated monitoring network capable of supporting conservation efforts, informing shipping route regulations and enabling timely responses to ecological disturbances. Moreover, by coupling detection certainty with spatial context, the system could prioritize and filter detections, further reducing false positives and optimizing researcher attention and computational resources. Ultimately, the combination of intelligent classification with spatial inference would push the field closer to autonomous, high-resolution ecological surveillance in challenging underwater environments.

## Figures and Tables

**Table 1:** Summary of dataset size and class distribution for the augmented dataset.

Split	Humpback	No Call	Total
Training	17,083	11,049	28,132
Validation	3312	2724	6036
Test	270	1207	1477
**Total**	20,665	14,980	35,645

Composition of the augmented dataset using 70/15/15 source-based splitting.

**Table 2:** Summary of dataset size and class distribution for the non-augmented dataset.

Split	Humpback	No Call	Total
Training	3173	2287	5460
Validation	602	307	909
Test	739	680	1419
**Total**	4514	3274	7788

Composition of the non-augmented dataset using 70/15/15 source-based splitting.

**Table 3:** CNN mel spectrogram model evaluation.

Metric	Value
Test Accuracy (%)	98.92
Test Loss	0.08
False Negative Rate (FNR)	0.01
False Positive Rate (FPR)	0.03
Matthews Correlation Coefficient (MCC)	0.96

Overall performance metrics for the CNN trained on mel spectrogram inputs.

**Table 4:** Classification report for custom CNN model.

Class	Precision (%)	Recall (%)	F1-Score (%)	Support
Humpback Whale	97	97	97	270
No Call	99	99	99	1207
Accuracy (%)			99	1477
Macro Avg (%)	98	98	98	1477
Weighted Avg (%)	99	99	99	1477

Precision, recall, F1-score and support for each class.

**Table 5:** MobileNetV2 model evaluation.

Metric	Value
Test Accuracy (%)	98.10
Test Loss	0.06
False Negative Rate (FNR)	0.02
False Positive Rate (FPR)	0.00
Matthews Correlation Coefficient (MCC)	0.94

Overall evaluation metrics for the pretrained MobileNetV2 model.

**Table 6:** Classification report for mobileNetV2 model.

Class	Precision (%)	Recall (%)	F1-Score (%)	Support
Humpback Whale	91	100	95	270
No Call	100	98	99	1207
Accuracy (%)			98	1477
Macro Avg (%)	95	99	97	1477
Weighted Avg (%)	98	98	98	1477

Precision, recall, F1-score, and class support for the pretrained MobileNetV2 model.

**Table 7:** CNN mel spectrogram model rvaluation (no augmentation).

Metric	Value
Test Accuracy (%)	96.05
Test Loss	0.13
False Negative Rate (FNR)	0.00
False Positive Rate (FPR)	0.08
Matthews Correlation Coefficient (MCC)	0.92

Overall performance metrics for the CNN trained on mel spectrogram inputs without augmentation.

**Table 8:** Classification report for CNN mel spectrogram model (no augmentation).

Class	Precision (%)	Recall (%)	F1-Score (%)	Support
Humpback Whale	100	92	96	739
No Call	92	100	96	680
Accuracy (%)			96	1419
Macro Avg (%)	96	96	96	1419
Weighted Avg (%)	96	96	96	1419

Precision, recall, F1-score, and support for each class.

**Table 9:** MobileNetV2 model evaluation (no augmentation).

Metric	Value
Test Accuracy (%)	99.01
Test Loss	0.03
False Negative Rate (FNR)	0.01
False Positive Rate (FPR)	0.01
Matthews Correlation Coefficient (MCC)	0.98

Overall performance metrics for the pretrained MobileNetV2 model without augmentation.

**Table 10:** Classification report for mobileNetV2 model (no augmentation).

Class	Precision (%)	Recall (%)	F1-Score (%)	Support
Humpback Whale	99	99	99	739
No Call	99	99	99	680
Accuracy (%)			99	1419
Macro Avg (%)	99	99	99	1419
Weighted Avg (%)	99	99	99	1419

Precision, recall, F1-score, and class support for the pretrained MobileNetV2 model without augmentation.

**Table 11:** CNN MFCC model evaluation (with augmentation).

Metric	Value
Test Accuracy (%)	87.27
Test Loss	0.42
False Negative Rate (FNR)	0.15
False Positive Rate (FPR)	0.02
Matthews Correlation Coefficient (MCC)	0.70

Overall performance metrics for the CNN trained on MFCC inputs with augmentation.

**Table 12:** Classification report for CNN MFCC model (with augmentation).

Class	Precision (%)	Recall (%)	F1-Score (%)	Support
Humpback Whale	59	98	74	270
No Call	100	85	92	1207
Accuracy (%)			87	1477
Macro Avg (%)	79	91	83	1477
Weighted Avg (%)	92	87	88	1477

Precision, recall, F1-score, and support for the CNN trained on MFCC inputs with augmentation.

**Table 13:** Pretrained MobileNetV2 MFCC model evaluation.

Metric	Value
Test Accuracy (%)	92.55
Test Loss	0.22
False Negative Rate (FNR)	0.09
False Positive Rate (FPR)	0.03
Matthews Correlation Coefficient (MCC)	0.80

Overall performance metrics for the pretrained MobileNetV2 model trained on MFCC inputs with augmentation.

**Table 14:** Classification report for pretrained MobileNetV2 MFCC model.

Class	Precision (%)	Recall (%)	F1-Score (%)	Support
Humpback Whale	72	97	83	270
No Call	99	91	95	1207
Accuracy (%)			93	1477
Macro Avg (%)	86	94	89	1477
Weighted Avg (%)	94	93	93	1477

Precision, recall, F1-score, and support for the pretrained MobileNetV2 MFCC model.

**Table 15:** CNN MFCC model evaluation (no augmentation).

Metric	Value
Test Accuracy (%)	96.05
Test Loss	0.16
False Negative Rate (FNR)	0.08
False Positive Rate (FPR)	0.00
Matthews Correlation Coefficient (MCC)	0.92

Overall performance metrics for the CNN trained on MFCC inputs without augmentation.

**Table 16:** Classification report for CNN MFCC model (no augmentation).

Class	Precision (%)	Recall (%)	F1-Score (%)	Support
Humpback Whale	93	100	96	739
No Call	100	92	96	680
Accuracy (%)			96	1419
Macro Avg (%)	96	96	96	1419
Weighted Avg (%)	96	96	96	1419

Precision, recall, F1-score, and support for the CNN MFCC model without augmentation.

**Table 17:** Pretrained MFCC model evaluation (no augmentation).

Metric	Value
Test Accuracy (%)	92.60
Test Loss	0.19
False Negative Rate (FNR)	0.08
False Positive Rate (FPR)	0.07
Matthews Correlation Coefficient (MCC)	0.85

Overall performance metrics for the pretrained CNN using MFCC inputs without augmentation.

**Table 18:** Classification report for pretrained MFCC model (no augmentation).

Class	Precision (%)	Recall (%)	F1-Score (%)	Support
Humpback Whale	93	93	93	739
No Call	92	92	92	680
Accuracy (%)			93	1419
Macro Avg (%)	93	93	93	1419
Weighted Avg (%)	93	93	93	1419

Precision, recall, F1-score, and support for the pretrained model using MFCC inputs without augmentation.

**Table 19:** Custom ViT model evaluation (with augmentation).

Metric	Value
Test Accuracy (%)	97.97
Test Loss	0.09
False Negative Rate (FNR)	0.02
False Positive Rate (FPR)	0.03
Matthews Correlation Coefficient (MCC)	0.93

Overall performance metrics for the custom ViT trained with augmentation.

**Table 20:** Classification report for custom ViT model (with augmentation).

Class	Precision (%)	Recall (%)	F1-Score (%)	Support
Humpback Whale	92	97	95	270
No Call	99	98	99	1207
Accuracy (%)			98	1477
Macro Avg (%)	96	98	97	1477
Weighted Avg (%)	98	98	98	1477

Precision, recall, F1-score, and support for the custom ViT model trained with augmentation.

**Table 21:** Custom ViT model evaluation (mo augmentation).

Metric	Value
Test Accuracy (%)	82.80
Test Loss	0.36
False Negative Rate (FNR)	0.35
False Positive Rate (FPR)	0.01
Matthews Correlation Coefficient (MCC)	0.69

Overall performance metrics for the custom ViT trained without augmentation.

**Table 22:** Classification report for custom ViT model (no augmentation).

Class	Precision (%)	Recall (%)	F1-Score (%)	Support
Humpback Whale	76	99	86	739
No Call	98	65	78	680
Accuracy (%)			83	1419
Macro Avg (%)	87	82	82	1419
Weighted Avg (%)	86	83	82	1419

Precision, recall, F1-score, and support for the custom ViT model trained without augmentation.

**Table 23:** Custom ViT MFCC model evaluation (with augmentation).

Metric	Value
Test Accuracy (%)	93.36
Test Loss	0.17
False Negative Rate (FNR)	0.07
False Positive Rate (FPR)	0.04
Matthews Correlation Coefficient (MCC)	0.81

Overall performance metrics for the Custom ViT trained on MFCCs with augmentation.

**Table 24:** Classification report for custom ViT MFCC model (with augmentation).

Class	Precision (%)	Recall (%)	F1-Score (%)	Support
Humpback Whale	75	96	84	270
No Call	99	93	96	1207
Accuracy (%)			93	1477
Macro Avg (%)	87	94	90	1477
Weighted Avg (%)	95	93	94	1477

Precision, recall, F1-score, and support for the Custom ViT trained on augmented MFCC inputs.

**Table 25:** Custom ViT MFCC model evaluation (no augmentation).

Metric	Value
Test Accuracy (%)	94.08
Test Loss	0.18
False Negative Rate (FNR)	0.06
False Positive Rate (FPR)	0.05
Matthews Correlation Coefficient (MCC)	0.88

Overall performance metrics for the Custom ViT trained on MFCC inputs without augmentation.

**Table 26:** Classification report for custom ViT MFCC model (no augmentation).

Class	Precision (%)	Recall (%)	F1-Score (%)	Support
Humpback Whale	94	95	94	739
No Call	94	94	94	680
Accuracy (%)			94	1419
Macro Avg (%)	94	94	94	1419
Weighted Avg (%)	94	94	94	1419

Precision, recall, F1-score, and support for the Custom ViT trained on MFCC inputs without augmentation.

**Table 27:** Comparison of all models across feature types and augmentation settings.

Model	Input	Aug?	Accuracy (%)	Whale Precision (%)	Whale Recall (%)	MCC
Custom CNN	Mel	Yes	98.92	97	97	0.96
Custom CNN	Mel	No	96.05	100	92	0.92
Custom CNN	MFCC	Yes	87.27	59	98	0.70
Custom CNN	MFCC	No	96.05	93	100	0.92
MobileNetV2 (Pretrained)	Mel	Yes	98.10	91	100	0.94
MobileNetV2 (Pretrained)	Mel	No	99.01	99	99	0.98
MobileNetV2 (Pretrained)	MFCC	Yes	92.55	72	97	0.80
MobileNetV2 (Pretrained)	MFCC	No	92.60	93	93	0.85
Custom ViT	Mel	Yes	97.97	92	97	0.93
Custom ViT	Mel	No	82.80	76	99	0.69
Custom ViT	MFCC	Yes	93.36	75	96	0.81
Custom ViT	MFCC	No	94.08	94	95	0.88

Unified comparison of all architectures trained on mel spectrograms or MFCC features, with and without augmentation.

**Table 28:** False positive and false negative rates across all models.

Model	Input	Aug?	FPR (%)	FNR (%)
CNN	Mel	Yes	2.59	0.75
CNN	Mel	No	7.58	0.00
CNN	MFCC	Yes	1.85	15.16
CNN	MFCC	No	0.14	8.09
MobileNetV2 (Pretrained)	Mel	Yes	0.00	2.32
MobileNetV2 (Pretrained)	Mel	No	0.68	1.32
MobileNetV2 (Pretrained)	MFCC	Yes	2.59	8.53
MobileNetV2 (Pretrained)	MFCC	No	7.04	7.79
Custom ViT	Mel	Yes	2.96	1.82
Custom ViT	Mel	No	1.08	34.71
Custom ViT	MFCC	Yes	4.07	7.21
Custom ViT	MFCC	No	5.41	6.47

**Table 29:** Comparison with existing whale detection and classification studies.

Study	Task	Feat.	Acc. (%)	F1 (%)	AUC
[37]	Benchmark/multi	Various	–	79	–
[38]	Binary	Mel+PCEN	–	97	0.992
[39]	Multi-species	Spec./MFCC	–	95	–
[40]	4-class	MFCC	95.0	95	0.98
[41]	Binary	Spec.+C-ViT	97.25	97	–
[42]	Binary	Wavelet	89.4	89	–
Custom CNN	Binary	Mel	98.92	99	0.992
MobileNetV2	Binary	Mel	99.01	98	0.9996

Note: “–” indicates metrics not reported in the respective studies, PCEN stands for per-channel energy normalization.

## Data Availability

The original contributions presented in this study are included in the article. Further inquiries can be directed to the corresponding author.

## Abbreviations

The following abbreviations are used in this manuscript:

CNN	Convolutional Neural Network
ViT	Vision Transformer
MFCC	Mel-Frequency Cepstral Coefficients
AODN	Australian Ocean Data Network

## References

[1] Mutanu L., Gohil J., Gupta K., Wagio P., Kotonya G. (2022). A review of automated bioacoustics and general acoustics classification research. Sensors.

[2] Lou R., Lv Z., Dang S., Su T., Li X. (2023). Application of machine learning in ocean data. Multimed. Syst..

[3] Mishachandar B., Vairamuthu S. (2021). Diverse ocean noise classification using deep learning. Appl. Acoust..

[4] Dommergues B., Cruz E., Vaz G. (2022). Optimization of underwater acoustic detection of marine mammals and ships using CNN. Proc. Meet. Acoust..

[5] Targ S., Almeida D., Lyman K. (2016). Resnet in Resnet: Generalizing residual architectures. arXiv.

[6] Montgomery J.C., Radford C.A. (2017). Marine bioacoustics. Curr. Biol..

[7] Ruff Z.J., Lesmeister D.B., Appel C.L., Sullivan C.M. (2021). Workflow and convolutional neural network for automated identification of animal sounds. Ecol. Indic..

[8] Dunlop R.A., Cato D.H., Noad M.J. (2008). Non-song acoustic communication in migrating humpback whales (*Megaptera novaeangliae*). Mar. Mammal Sci..

[9] Pace F., Benard F., Glotin H., Adam O., White P. (2010). Subunit definition and analysis for humpback whale call classification. Appl. Acoust..

[10] Dunlop R.A. (2019). The effects of vessel noise on the communication network of humpback whales. R. Soc. Open Sci..

[11] Mann D., Locascio J., Wall C., Au W.W.L., Lammers M.O. (2016). Listening in the ocean: New discoveries and insights on marine life from autonomous passive acoustic recorders. Listening in the Ocean.

[12] Oppenheim A.V. (1970). Speech spectrograms using the fast Fourier transform. IEEE Spectr..

[13] Palanisamy K., Singhania D., Yao A. (2020). Rethinking CNN models for audio classification. arXiv.

[14] Seo S., Kim C., Kim J.-H. (2022). Convolutional neural networks using log Mel-spectrogram separation for audio event classification with unknown devices. J. Web Eng..

[15] Meng H., Yan T., Yuan F., Wei H. (2019). Speech emotion recognition from 3D log-Mel spectrograms with deep learning network. IEEE Access.

[16] Cotton C.V., Ellis D.P.W. Spectral vs. spectro-temporal features for acoustic event detection. Proceedings of the 2011 IEEE Workshop on Applications of Signal Processing to Audio and Acoustics (WASPAA).

[17] Valero X., Alias F. (2012). Gammatone Cepstral Coefficients: Biologically Inspired Features for Non-Speech Audio Classification. IEEE Trans. Multimed..

[18] Anagnostou M.N., Nystuen J.A., Anagnostou E.N., Papadopoulos A., Lykousis V. (2011). Passive aquatic listener (PAL): An adoptive underwater acoustic recording system for the marine environment. Nucl. Instrum. Methods Phys. Res. A.

[19] Roh Y., Heo G., Whang S.E. (2021). A survey on data collection for machine learning: A big data—AI integration perspective. IEEE Trans. Knowl. Data Eng..

[20] Open Access to Ocean Data. https://portal.aodn.org.au/.

[21] Ocean Alliance Ocean Alliance—Whale Research & Conservation. https://whale.org/.

[22] BBC Sound Effects. https://sound-effects.bbcrewind.co.uk/search.

[23] Watkins Marine Mammal Sound Database. https://whoicf2.whoi.edu/science/B/whalesounds/index.cfm.

[24] Eklund V.-V. Data Augmentation Techniques for Robust Audio Analysis. https://trepo.tuni.fi/handle/10024/117251.

[25] Mushtaq Z., Su S.-F., Tran Q.-V. (2021). Spectral images based environmental sound classification using CNN with meaningful data augmentation. Appl. Acoust..

[26] Wei S., Zou S., Liao F., Lang W. (2020). A comparison on data augmentation methods based on deep learning for audio classification. J. Phys. Conf. Ser..

[27] Hwang Y., Cho H., Yang H., Won D.-O., Oh I., Lee S.-W. (2020). Mel-spectrogram augmentation for sequence to sequence voice conversion. arXiv.

[28] Nanni L., Maguolo G., Paci M. (2020). Data augmentation approaches for improving animal audio classification. Ecol. Inform..

[29] Salamon J., Bello J.P. (2017). Deep Convolutional Neural Networks and Data Augmentation for Environmental Sound Classification. IEEE Signal Process. Lett..

[30] Imran M.S., Rahman A.F., Tanvir S., Kadir H.H., Iqbal J., Mostakim M. An analysis of audio classification techniques using deep learning architectures. Proceedings of the 2021 6th International Conference on Inventive Computation Technologies (ICICT).

[31] Alsaleh A., Perkgoz C. (2023). A space and time efficient convolutional neural network for age group estimation from facial images. PeerJ Comput. Sci..

[32] Wei D., Li J., Pham P., Das S., Qu S. Acoustic scene recognition with deep neural networks (DCASE challenge 2016). Proceedings of the Detection and Classification of Acoustic Scenes and Events 2016.

[33] Maurício J., Domingues I., Bernardino J. (2023). Comparing vision transformers and convolutional neural networks for image classification: A literature review. Appl. Sci..

[34] Reedha R., Dericquebourg E., Canals R., Hafiane A. (2022). Transformer neural network for weed and crop classification of high resolution UAV images. Remote Sens..

[35] Dosovitskiy A., Beyer L., Kolesnikov A., Weissenborn D., Zhai X., Unterthiner T., Dehghani M., Minderer M., Heigold G., Gelly S. (2021). An Image is worth 16x16 words: Transformers for image recognition at scale. arXiv.

[36] Kowarski K.A., Moors-Murphy H. (2021). A review of big data analysis methods for baleen whale passive acoustic monitoring. Mar. Mamm. Sci..

[37] Schall E., Kaya I.I., Debusschere E., Devos P., Parcerisas C. (2024). Deep learning in marine bioacoustics a benchmark for baleen whale detection. Remote Sens. Ecol. Conserv..

[38] Allen A.N., Harvey M., Harrell L., Jansen A., Merkens K.P., Wall C.C., Cattiau J., Oleson E.M. (2021). A convolutional neural network for automated detection of humpback whale song in a diverse, long-term passive acoustic dataset. Front. Mar. Sci..

[39] Shiu Y., Palmer K.J., Roch M.A., Fleishman E., Liu X., Nosal E.-M., Helble T., Cholewiak D., Gillespie D., Klinck H. (2020). Deep neural networks for automated detection of marine mammal species. Sci. Rep..

[40] Raza A., Zongxin S., Qiao G., Javed M., Bilal M., Zuberi H.H., Mohsin M. (2025). Automated classification of humpback whale calls in four regions using convolutional neural networks and multi-scale deep feature aggregation (MSDFA). Measurement.

[41] Masruroh S.U., Junas M.D., Hulliyah K., Sukmana H.T., Putri R.A., Aripiyanto S. Convolutional vision transformer modeling for spectrogram image processing in the detection of North Atlantic right whales up-call. Proceedings of the 2024 5th International Conference on Big Data Analytics and Practices (IBDAP).

[42] Babalola O.P., Usman A.M., Odeyemi S.G., Rufai K.I. (2024). Wavelet based feature extraction with hidden Markov model classification of Antarctic blue whale sounds. Ecol. Inform..

